# Parental investment and body temperature explain encephalization in vertebrates

**DOI:** 10.1073/pnas.2506145122

**Published:** 2025-11-03

**Authors:** Zitan Song, Michael Griesser, Carel P. van Schaik

**Affiliations:** ^a^Comparative Socioecology Group, Department for the Ecology of Animal Societies, Max Planck Institute of Animal Behavior, Konstanz 78467, Germany; ^b^Department of Biology, University of Konstanz, Konstanz 78467, Germany; ^c^Centre for the Advanced Study of Collective Behaviour, University of Konstanz, Konstanz 78467, Germany; ^d^Department of Collective Behavior, Max Planck Institute of Animal Behavior Konstanz, Konstanz 78467, Germany; ^e^Department of Evolutionary Anthropology, University of Zurich, Zurich 8057, Switzerland; ^f^Institute for the Interdisciplinary Study of Language Evolution, University of Zurich, Zurich 8050, Switzerland

**Keywords:** expensive brain hypothesis, prehatching provisioning, parental investment, body temperature, newborn size

## Abstract

The systematic variation in relative brain size among vertebrate classes remains poorly understood. Here, based on the expensive brain hypothesis, we propose that two broad constraints explain much of the variation: 1) the ability to produce large offspring, and so provide them with the energy required for constructing larger brains, and 2) the ability to sustain continuously high body temperatures, because cooler and varying brain temperatures reduce brain performance and thus fitness. We therefore predicted that encephalization (major evolutionary increases in brain size) only happened where changes in physiology or natural history created these abilities. First, comparative analyses across all major vertebrate classes (n = 2600 species) revealed that protecting or provisioning eggs or embryos is associated with larger newborns. Subsequent analyses at the class level confirmed that newborn size and adult brain size underwent correlated evolution in birds, mammals, and cartilaginous fishes, but not in other fishes, amphibians, and reptiles. Second, we found a positive relationship between mean body temperature and brain size within each class (albeit sometimes insignificant). Third, a combined analysis across all vertebrates revealed a positive interaction between the effects of body temperature and newborn size. In conclusion, encephalization became most pronounced in vertebrate lineages that can both produce large offspring, reflecting internal fertilization with matrotrophy, and sustain high body temperature, partly linked to endothermy.

## Significance

Larger brains generally improve sensory, motor, and cognitive functioning, but this does not explain why brain size shows marked variation across vertebrate classes and increased over evolutionary time in some classes but not others. This pattern suggests that brain size expansion is affected by taxonomy-dependent costs or constraints. These are the focus of the expensive brain hypothesis, for which evidence has already been found in some taxa. Here, we systematically test two of its main predictions across all vertebrate classes and identify two conditions that together provide a coherent theoretical explanation for the well-known pattern in vertebrates brain size: 1) opportunities for greater parental investment into individual offspring, and 2) opportunities for stably increased brain temperatures.

**Fig. 1.**
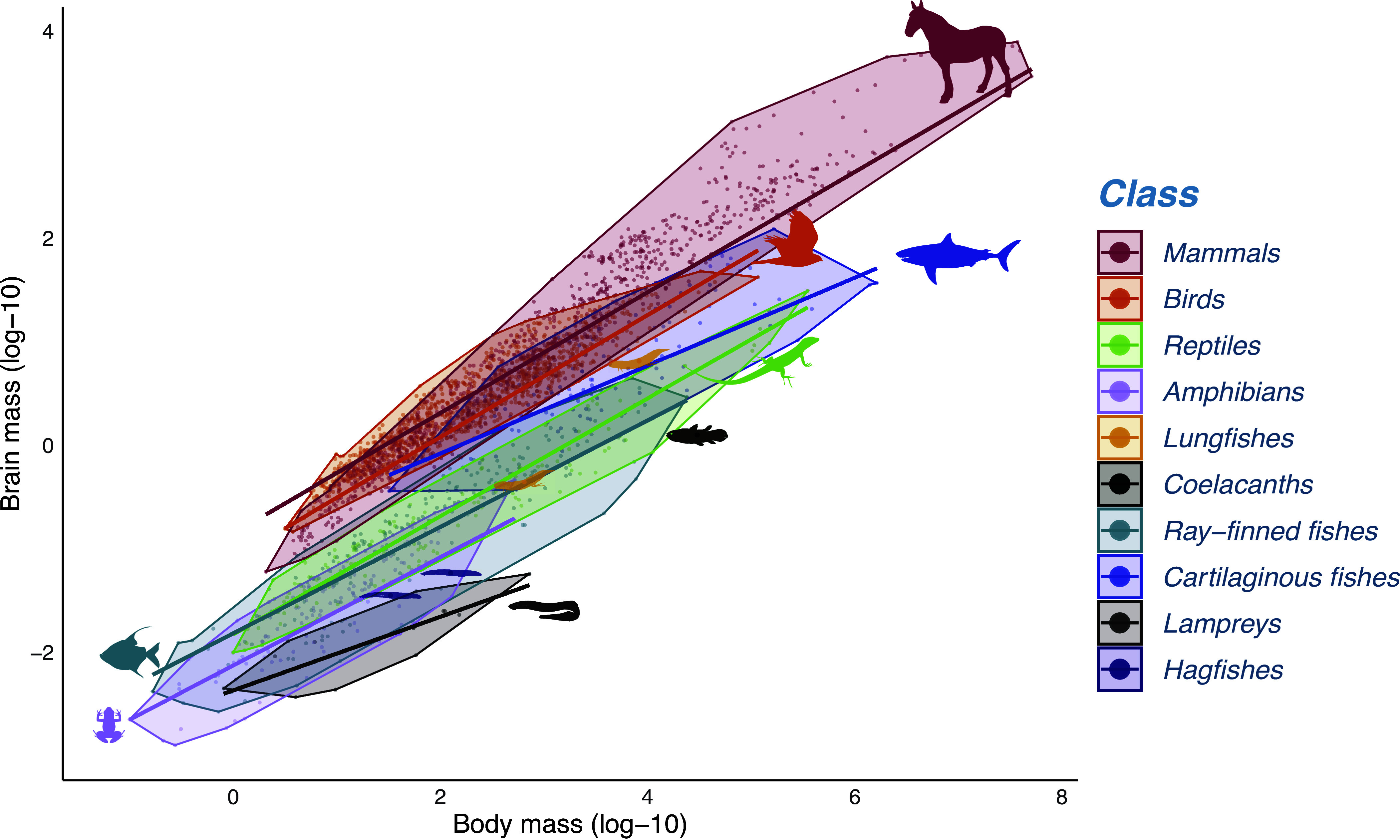
The relationship between brain mass and body mass across vertebrates, by class. The species in this plot are those included in this study. Please note that the symbols for lungfishes, coelacanths, and hagfishes represent individual species. The regression lines were generated using the *phylogenetic generalized least squares* models from the phylolm package (*SI Appendix,* Table S6).

Among vertebrates, relative brain sizes (i.e., controlling for body mass) vary by over two orders of magnitude, and more recently evolved classes have relatively larger brains than more ancestral ones (1, 2). One common explanation is that the evolution of endothermy promoted major brain size increases (e.g., refs. 3 and 4): for a given body size, the brain of the average bird or mammal has become about 10 times larger than of an ectothermic vertebrate. However, endothermy alone cannot explain this pattern (Fig. 1). The ancient and predominantly ectothermic cartilaginous fishes (Chondrichthyes) have relative brain sizes similar to those of endothermic precocial birds (1, 5), whereas the other fish classes and amphibians have remained small-brained. Neither can the variation be readily attributed to variation in the cognitive benefits of larger brain size linked to ecological or social life, often suggested to explain brain size variation in endotherms [ecological brain or social brain (6)]. Socioecological pressures overlap between endotherms and ectotherms (7), yet even birds and mammals with the simplest diets and social systems have substantially larger brains relative to body size than fishes with more complex social systems or diets. The lack of a plausible explanation for the broad pattern of vertebrate brain size variation suggests that other, as yet unidentified, factors are also at play.

Here, extending earlier suggestions (5, 8), we examine the expensive brain hypothesis (9–11). It argues that the brain’s unusually high and especially constant energy requirements (9, 12, 13) can limit its size despite potential fitness benefits of increased brain size due to cognitive or sensorimotor adaptations. This hypothesis is empirically well-supported: energy constraints are known to limit brain size in some endotherms (14), as do lower body temperature in some ectotherms (15). The expensive brain hypothesis implies that if selection favors encephalization (i.e., a marked evolutionary increase in relative brain size), the organism must be able to meet the disproportionate rise in energy requirements. The overall aim of this paper is to explain brain size variation among vertebrates based on two predictions arising from this hypothesis, the first one concerning the brain size correlates of the size of hatchlings or neonates (henceforth: newborns) and the second one concerning body temperature.

First, we invoke a developmental version of the expensive brain hypothesis, known as the parental provisioning hypothesis (16, 17). It proposes that variation in parental investment in offspring, both before (5) and after (17, 18) hatching or birth, explains much of the variation in brain size that evolved among and within vertebrate lineages. Brains are especially costly for developing young, because selection generally favors high somatic growth rates early in life to minimize the period of greatest vulnerability (19, 20). The ensuing competition between the growth of the body and the brain can hinder the evolution of larger-brained organisms that must learn and practice various vital foraging or antipredation skills (21, 22). The head-start needed to grow a larger brain would therefore require higher parental investment and thus larger young. Accordingly, the parental provisioning hypothesis posits that greater parental investment will alleviate developmental obstacles to encephalization (16, 17). This hypothesis is consistent with the finding that rates of brain growth relative to body size during development are much higher during the time of active parental investment (8), and that species with larger offspring at birth generally have larger brains, as observed in cartilaginous fishes (5), some cichlids (23), and mammals (10). However, so far no systematic comparative study has tested the prediction that brain size underwent correlated evolution with parental provisioning, and thus offspring size, before or after birth or hatching, across all vertebrates.

The first aim of this paper is to comprehensively test this prediction in two steps. In step A, we identify the conditions under which parents can produce larger newborns. This step is required because the coevolution of newborn size and brain size is prevented if there is a fundamental limit to offspring size. According to classic life-history theory, allocating a given amount of energy to fewer, larger offspring is only favored by selection if the increase in parental fitness due to higher survival of the young outweighs the reduction due to lower fertility (20, 24, 25). During the prenatal phase, organisms with external fertilization can increase offspring survival by protecting eggs prehatching (23), while species with internal fertilization do so automatically (cf., ref. 5). These actions permit the production of bigger offspring. We categorized behaviors that affect egg or embryo survival in ascending order of protection and provisioning (details in *SI Appendix,* Table S1): i) abandoning eggs, ii) guarding eggs after deposition, iii) bearing eggs, and iv) provisioning eggs (matrotrophy). Thus, we tested whether increased protection and provisioning of eggs is associated with larger newborns. In step B we then tested the actual prediction that newborn size was correlated with adult brain size, in classes with enough species to permit the analysis (*SI Appendix,* Table S2).

Our second aim was to test the other prediction of the expensive brain hypothesis on encephalization, which concerns the role of body temperature. Ectothermic animals living in constantly or periodically cold environments may not benefit from encephalization, particularly when they are small as adults. First, although overall metabolism in active ectotherms goes down as body temperature decreases due to the temperature dependence of biochemical processes (26), energy use by the brain decreases far less (23, 27). This suggests that maintaining the brain’s functionality at lower body temperatures requires energetically costly biochemical adaptations, as shown by the high energy costs of acclimatizing to lower temperatures (28). Thus, as body temperature goes down, brains consume an ever-larger share of the organism’s energy budget, which makes them relatively more expensive. Second, the large fluctuations in body temperature of heterothermic animals negatively affect brain processing ability (29), and presumably learning (30), further reducing the fitness benefits of increased brain size at low temperatures. We therefore predicted that species with cooler or more fluctuating body temperatures did not encephalize, even if they produce larger newborns.

Because body temperature is linked to overall metabolism (26), an alternative hypothesis consistent with the expensive brain framework, would be that metabolic rate directly influences brain size by affecting the energy available to the brain (31, 32); but see refs. 33 and 34, although none of these studies controlled for the effect of body temperature. A number of variables, such as activity period (15), overall activity level, and especially ambient temperature (35) may weaken the effect of metabolic rate on energy allocation to the brain. In addition, life-history strategy may also independently affect it (10). Finally, basking (found in a few mammals, cartilaginous fishes, and amphibians, but especially reptiles) may weaken this link even more. It should therefore be possible to disentangle the effects of body temperature and metabolic rate. Thus, we also directly compared the power of this alternative hypothesis and the body temperature hypothesis to predict brain size, while controlling for a possible confounding effect of ambient temperature.

The tests of newborn size and mean body temperature on brain size were conducted separately to maximize the number of species available in each analysis (n = 2,580 and 1,059, respectively), but we also used the smaller sample to test for the combined effect of newborn size and body temperature. In all analyses, we used phylogenetically controlled linear mixed models in the R package MCMCglmm including brain size, body size, and newborn size, and where relevant, additional predictors. *SI Appendix,* Table S2 lists the number of species per class included in each analysis.

## Results

### Prehatching Provisioning and Brain Size.

**Fig. 2.**
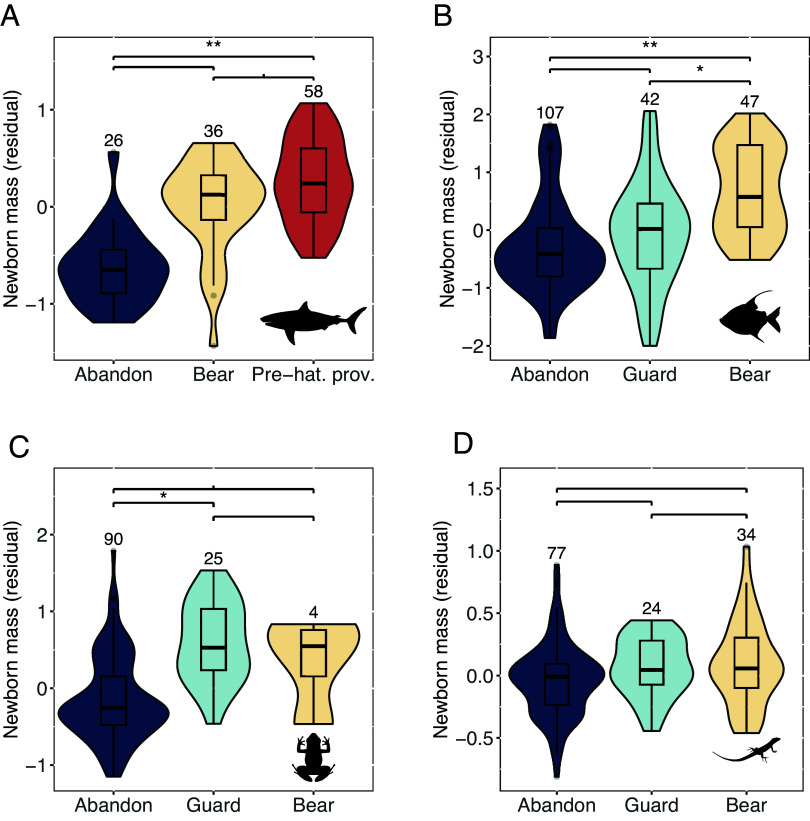
Effects of level of care and provisioning of eggs on the size of newborns. (*A*–*D*) Comparisons between the relative newborn sizes (illustrated here as residual values) in relation to egg care category among cartilaginous fishes (*A*), ray-finned fishes (*B*), amphibians (*C*), and reptiles (*D*). The number of species analyzed is indicated above each violin plot. For statistics see *SI Appendix,* Table S3.

We first tested the expectation of life-history theory that species showing a higher level of egg care (protection or provisioning) would produce larger-bodied newborns relative to parental body size within each vertebrate class with variable egg care. We confirmed this prediction in cartilaginous fishes, ray-finned fishes, and amphibians, but not in reptiles (Fig. 2; see *SI Appendix,* Table S3 for the statistical model). Because almost all birds and all mammals provision their eggs, we cannot examine the effect of variation in egg care levels within each class. Nonetheless, their ratios of offspring mass to adult mass are among the highest in vertebrates (*SI Appendix,* Fig. S1), consistent with the prediction. Overall, there was a clear association between higher care levels and larger relative newborn size. Hence, the precondition for coevolution between newborn size and brain size is met.

**Fig. 3.**
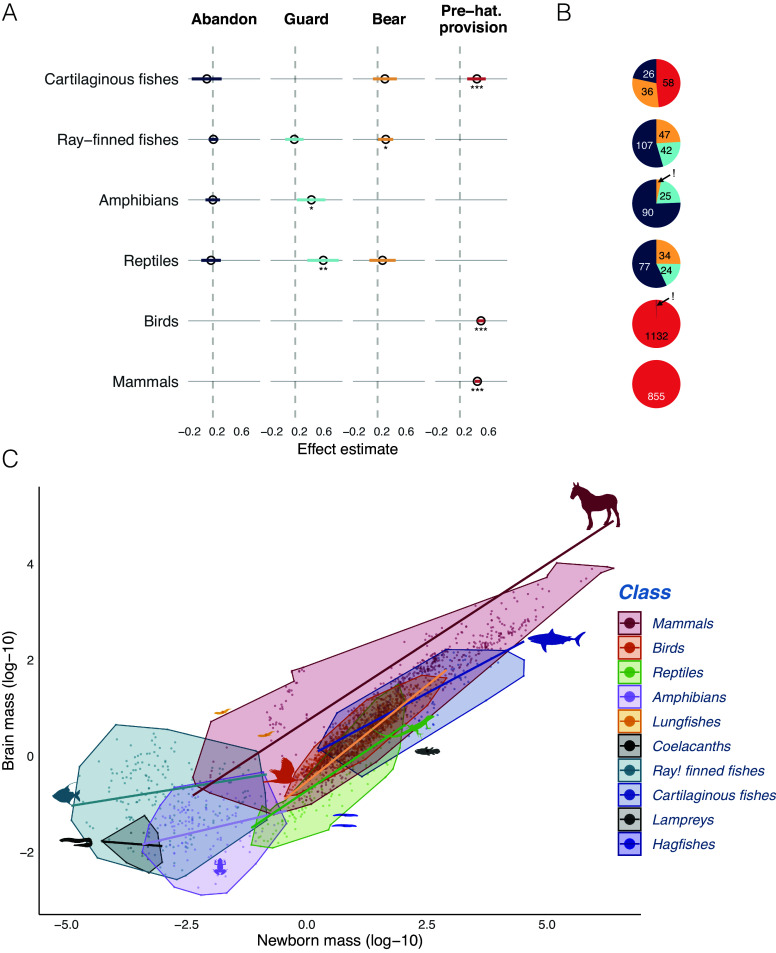
Newborn size and brain size among vertebrate classes. (*A*) Forest plot of the effect of ectothermic prehatching investment (newborn size) on adult brain size (controlled by body mass) for a given category of egg care, for each of the classes with an adequate sample size. (*B*) The distribution across species in the sample of the various egg care categories (see Fig. 3*A* for color codes). (*C*) Adult brain size vs newborn size relationships within and among classes. The regression lines in *C* were generated using the phylogenetic generalized least squares model from the phylolm package (*SI Appendix,* Table S5).

Next, we asked if adult brain size coevolved with newborn size, when controlling for adult body size. As predicted, when species merely abandon their eggs, there was no correlated evolution between brain size and newborn size in each of the four ectothermic classes tested (Fig. 3 *A* and *B* and *SI Appendix,* Table S4). However, within specific care levels, brain size increased with offspring size, as predicted (Fig. 3*A* and *SI Appendix,* Table S4). This pattern was uncovered among egg-bearing ray-finned fishes and egg-guarding reptiles and amphibians, as well as among the ectotherms engaging in the strongest form of care, prenatal provisioning: cartilaginous fishes [expanding upon (5)], (incubating) birds, and mammals. This pattern confirms the key prediction of the expensive brain hypothesis: brain size and the size of newborns only coevolved where there is some level of parental care of eggs, especially prehatching or prenatal provisioning. Note that the effect of postnatal care in mammals and altricial birds is not included here.

The combined effects of both care level, body size, and taxonomic class produced the following taxonomic pattern in adult brain size in relation to newborn mass (Fig. 3*C*). When controlling for body size, adult brain size was positively associated with newborn size in birds, mammals, and cartilaginous fishes, but not in the other classes (Table 1). As shown in Fig. 3*C*, the regression slopes of brain mass on offspring mass are steep in cartilaginous fishes, birds, and mammals, but far shallower in lampreys, ray-finned fishes, and amphibians (*SI Appendix,* Table S5). Although the regression slopes of brain mass on newborn mass in reptiles were close to birds and mammals, there was no significant effect of relative newborn mass on relative brain size (perhaps due to the temperature effect examined next).

**Table 1.** Phylogenetically controlled mixed model in the R package MCMCglmm assessing the effect of newborn mass on brain mass in each of the vertebrate classes with adequate sample size

	Posterior mean	Lower ▪ upper 95% CI	Effective sample size	*P* MCMC
**Lampreys (n = 14, λ = 0.945 (0.615, 1.000))**				
Intercept	0.019	−2.169 ▪ 2.322	10,000	0.992
**Body (log-10)**	**0.873**	**0.631 ▪ 1.114**	**10,000**	<0.001***
Newborn mass (log-10)	0.158	−0.241 ▪ 0.531	8,565	0.447
**Cartilaginous fishes (n = 120, λ = 0.961 (0.855, 0.999))**				
Intercept	−0.156	−0.743 ▪ 0.537	10,000	0.626
**Body (log-10)**	**0.619**	**0.537 ▪ 0.700**	**8,870**	<0.001***
**Newborn mass (log-10)**	**0.183**	**0.075 ▪ 0.286**	**1,0000**	<0.001***
**Ray-finned fishes (n = 210, λ = 0.976 (0.951, 0.991))**				
Intercept	−0.082	−0.652 ▪ 0.460	10,463	0.780
**Body (log-10)**	**0.788**	**0.742 ▪ 0.832**	**10,000**	<0.001***
Newborn mass (log-10)	0.014	−0.036 ▪ 0.067	10,000	0.581
**Amphibians (n = 130, λ = 0.726 (0.361, 0.927))**				
Intercept	−0.150	−0.705 ▪ 0.391	9,739	0.558
**Body (log-10)**	**0.723**	**0.622 ▪ 0.826**	**10,000**	<0.001***
Newborn mass (log-10)	0.056	−0.050 ▪ 0.162	9,690	0.291
**Reptiles (n = 190, λ = 0.918 (0.813, 0.984))**				
Intercept	0.025	−0.283 ▪ 0.327	10,000	0.878
**Body (log-10)**	**0.934**	**0.840 ▪ 1.026**	**10,000**	<0.001***
Newborn mass (log-10)	0.082	−0.003 ▪ 0.173	10,000	0.069
**Birds (n = 1,136, λ = 0.930 (0.910, 0.948))**				
Intercept	−0.399	−0.637 ▪ −0.156	10,000	<0.001
**Body (log-10)**	**0.727**	**0.675 ▪ 0.781**	**10,000**	<0.001***
**Newborn mass (log-10)**	**0.341**	**0.281 ▪ 0.400**	**10,000**	<0.001***
**Mammals (n = 855, λ = 0.972 (0.963, 0.980))**				
Intercept	0.178	−0.116 ▪ 0.475	9,661	0.240
**Body (log-10)**	**0.576**	**0.541 ▪ 0.612**	**10,000**	<0.001***
**Newborn mass (log-10)**	**0.286**	**0.245 ▪ 0.330**	**10,000**	<0.001***

λ: posterior phylogenetic signal (mean and 95% credible interval) from MCMCglmm.

Significant predictors are highlighted in bold.

### Body Temperature, Newborn Size, and Brain Size.

Although we found that higher care levels were associated with larger newborn size (Fig. 2) and larger brain size (Fig. 3*A*) in ray-fined fishes, amphibians, and reptiles, neither they nor the lampreys (which have only one egg care level) showed the expected effect of newborn size on adult brain size, and their brains are small overall (Fig. 1). Moreover, two other species-poor fish classes, hagfishes, and coelacanths, have remarkably large offspring at hatching (*SI Appendix,* Fig. S1), yet also have small brains (Fig. 1).

**Fig. 4.**
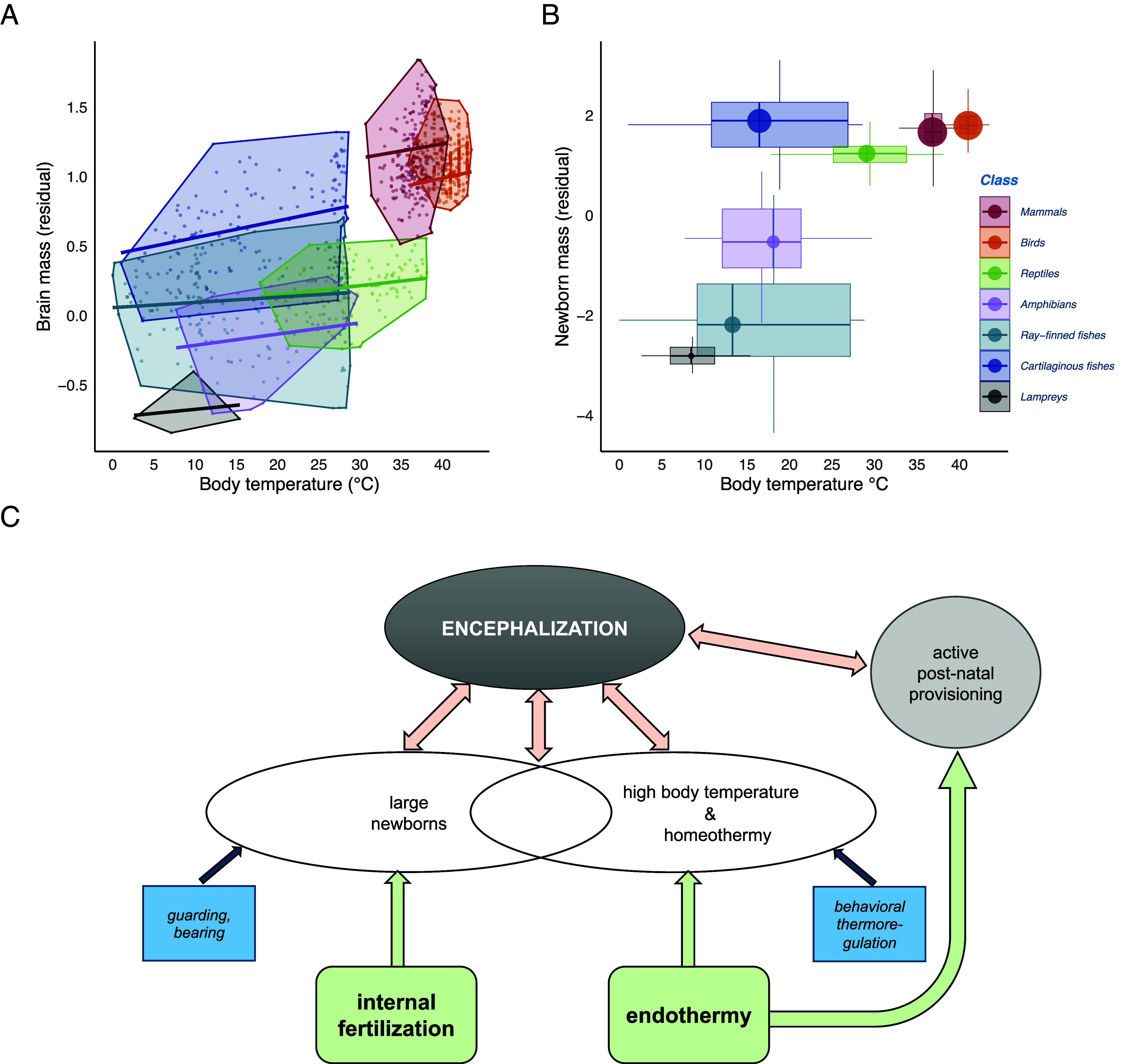
Temperature and brain size among vertebrate classes. (*A*) The relationship between relative brain mass (among all vertebrates) and body temperature across all vertebrate classes. The significance of the relationship was tested using scaled and centered relative brain mass values calculated for each class (*SI Appendix,* Table S7). (*B*) A visual rendition of the relationship between body temperature, relative newborn mass, and relative brain mass in all vertebrate classes. The vertical axis displays boxplots of relative newborn mass across all vertebrates, the horizontal axis boxplots of body temperature. The size of the circle, located at their joint median values, indicates the mean relative brain mass. (*C*) A conceptual diagram illustrating the main factors affecting vertebrate encephalization, as revealed by this study. Internal fertilization and endothermy were major independent facilitators of larger newborns and higher body temperatures, both of which subsequently underwent correlated evolution with brain size. Smaller, independent effects in ectotherms also affect newborn size and body temperature. Postnatal provisioning is not considered in these analyses.

To examine why no encephalization occurred in these classes, we tested (in a separate, smaller sample) the second prediction of the expensive brain hypothesis, namely a positive evolutionary relationship between brain size and mean body temperature (in fishes estimated as the modal water temperature, see Methods for details). When assessed within classes, all showed a positive relationship between mean body temperature and brain size, although this relationship was statistically significant only in the encephalized lineages: cartilaginous fishes, birds, and mammals (Fig. 4*A* and *SI Appendix,* Table S7), which also had relatively larger sample sizes.

When we assessed the effect of both newborn size and body temperature in a single analysis across all vertebrates, we found that both of these were significantly positively associated with brain size (Table 2). Importantly we also found a positive interaction between the two, indicating a synergistic effect between them (Table 2 and Fig. 4*B*).

**Table 2.** Phylogenetically controlled mixed model in the R package MCMCglmm assessing the effect of temperature and newborn mass on brain mass across all vertebrates (n = 1,059)

	Posterior Mean	Lower ▪ upper 95% CI	Effective sample size	*P* MCMC
Intercept	−0.582	−1.067 ▪ −0.086	9,679	0.020
**Body (log-10)**	**0.648**	**0.618 ▪ 0.675**	**10,000**	<0.001***
**Newborn mass (log-10, NM)**	**0.228**	**0.177 ▪ 0.283**	**10,000**	<0.001***
**Body temperature (Tb)**	**0.187**	**0.147 ▪ 0.227**	**10,000**	<0.001***
**NM × Tb**	**0.109**	**0.084 ▪ 0.134**	**10,000**	<0.001***

λ = 0.995 (0.993, 0.997).

Significant predictors are highlighted in bold.

**Fig. 5.**
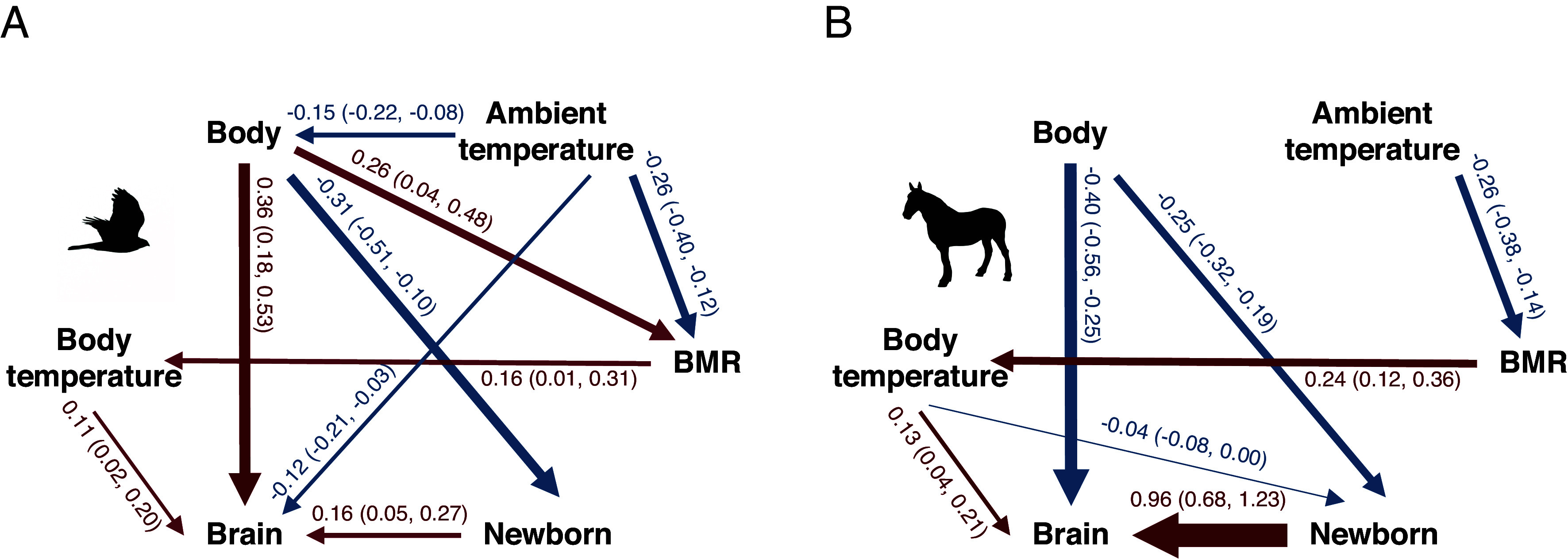
The structure of the relationships in the most parsimonious path models (*SI Appendix,* Table S9), for (*A*) birds and (*B*) mammals. Abbreviations: BMR = basal metabolic rate. Only significant relationships are shown, with mean and 95% CI added in parentheses; arrow thickness reflects significance level; arrow color the sign (blue = negative; red = positive).

Finally, we evaluated the alternative hypothesis proposing that metabolic rate rather than body temperature affects brain size. Thus, we conducted a phylogenetic path analysis to examine the causal relationships among residual newborn body size and adult body size, body temperature, ambient temperature, residual basal metabolic rate (BMR), and residual brain size in birds and mammals (the only two classes for which we had sufficient data). We tested 10 candidate models representing different scenarios linking BMR, body temperature, and ambient temperature to brain size (*SI Appendix,* Fig. S2). For mammals, a single best-supported model was identified; while for birds, two top-ranked models (ΔCICc < 2) were identified and subsequently averaged (*SI Appendix*, Table S8). The resulting model-averaged path coefficients are reported in Fig. 5 (see also *SI Appendix*, Table S9). In both cases, BMR did not have a direct effect on brain size. In birds, ambient temperature showed a negative effect on brain size, whereas in all cases brain size was primarily driven by body temperature and newborn body size, supporting the original model.

Because path models can only assess directional causality rather than coevolutionary relationships, we tested the robustness of these results with MCMCglmm models with residual brain size as the dependent variable and body mass, ambient temperature, body temperature, residual BMR, and residual newborn size as dependent ones. The results confirm the conclusions of the path analyses for both birds and mammals (*SI Appendix*, Table S10). Furthermore, because BMR is highly correlated with body size and ambient temperature, we also replaced residual BMR with a size- and temperature-adjusted metabolic rate quotient developed by Yegian et al. (36), which is among the most up-to-date interspecific standardizations of metabolic rate. The results remained consistent (*SI Appendix*, Table S10).

## Discussion

Our analyses confirmed two key preconditions for encephalization in vertebrates. First, where parents invest beyond merely depositing eggs, larger newborns can evolve, which enables encephalization. The most encephalized vertebrate classes (Fig. 1 and *SI Appendix,* Table S6) accordingly have the highest levels of care, namely provisioning of embryos: mammals (100% of species), birds (99%), cartilaginous fishes (48%). At the other extreme, the least encephalized classes in our sample abandon their eggs: lampreys (100%) and amphibians (75%). Ray-finned fishes and reptiles in our sample are intermediate in both care levels and brain size (Fig. 3*B*). Second, independently, higher body temperatures are associated with encephalization (Table 2 and Figs. 4 *A* and *B* and 5). Critically, these effects are synergistic, explaining the massive increase in brain size of birds and mammals.

These results remained robust when the body mass effect on brain size or newborn size was controlled for by using residual values from regression with body mass. This therefore dealt with two concerns: 1) the multicollinearity between newborn size and adult body size, and 2) the fact that newborn sizes in ectotherms could come from specimens with highly deviant adult body mass (*SI Appendix,* Tables S11–S15). Moreover, these results also remained robust against the alternative that the observed effect of body temperature was actually a direct reflection of metabolic rate, at least in birds and mammals. Phylogenetic path analysis (Fig. 5) showed that ambient temperature mainly affected BMR and had only a weak, negative effect on brain size in birds, whereas brain size remained most strongly influenced by body temperature. This result was confirmed by a MCMCglmm analysis (*SI Appendix*, Table S10). Unfortunately, this test was impossible for ectotherms due to lack of suitable data.

We generalized the earlier finding that parental protection and provisioning allows the production of fewer but bigger newborns (20, 24, 25). This result adds a reduced clutch size to the list of common life-history correlates of increased brain size (37–40). Examination of the role of postnatal parental provisioning was prevented by a lack of data on sizes of young at the end of parental investment in most endotherms and the few ectotherms engaging in it (41). Including it would certainly have strengthened the results. Indeed, in birds, altricial species, which engage in posthatching parental provisioning, are larger-brained than precocial ones, which do not (18, 42). Moreover, among altricial species, the duration of posthatching provisioning predicts brain size (18).

The evolution of larger newborns is facilitated by internal fertilization, which is therefore a major preadaptation [or exaptation sensu (43)] that facilitates encephalization (Fig. 4*C*). It is accordingly widespread in encephalized cartilaginous fishes. However, internal fertilization also occasionally evolved in ray-finned fishes (44), but there it did not increase brain size in our sample (*SI Appendix,* Table S16 and Fig. S3). Moreover, it is also very common in amphibians (45), which also are all small-brained. This pattern aligns with the finding that low body temperature limits brain size in these ectotherms.

We found that body temperature (directly measured during activity in tetrapods, estimated from modal water temperature in all fishes) was correlated with brain size across vertebrates. The current findings thus extend the explanatory reach of the expensive brain hypothesis. Periods of starvation select for both lower body temperatures, with (46) or without (47) being accompanied by torpor, and smaller brains, given that both serve to reduce the risk of falling into a negative energy balance (14, 15). Brains are less effective at lower body temperature anyway (15), a phenomenon also found in mammalian hibernators (15, 30, 48). Thus, where high and stable body temperatures are ecologically possible, this reduces the relative costs of brain size and thereby permit encephalization where that is adaptive.

Body temperature explained less variation in brain size relative to newborn size within the endotherms (Fig. 5, also see *SI Appendix*, Table S7), because interspecific variation in the mean and variance of body temperatures within them is generally very low [except in hibernators, which accordingly have smaller brains (48)]. For instance, although humans have clearly larger newborns, they do not have higher body temperature than our primate relatives. However, the temperature effect goes a long way to explaining the overall brain size difference between endotherms and ectotherms. Many ectotherms have body temperatures only marginally higher than ambient temperatures. Most amphibians are prevented from basking and so raising body temperature because their permeable skin would lead to rapid dehydration (49). Although many diurnal reptiles can bask and so raise their body temperatures well above ambient levels, at higher latitudes or altitudes this is possible only for a few hours per day (50). This strong heterothermy may prevent encephalization (15). The most informative case involves the cartilaginous fishes. They have larger brains than ray-finned fishes and tend to live in water that is on average around 3 °C warmer than ray-finned fishes do (*SI Appendix*, Fig. S4*A*). However, the use of water temperature to estimate body temperature for all fishes may have seriously underestimated it in cartilaginous fishes. In fact, the body temperature of many cartilaginous fishes can be up to 20 °C higher than the surrounding water (51), by a combination of being larger than the average ray-finned fish (*SI Appendix*, Fig. S4*B*), physiological adaptations (muscular heat production and countercurrent blood circulation), and behavioral thermoregulation (alternating deep dives with recovery times near the surface or migratory movements) (52, 53). Thus, the analyses underlying Fig. 4*B* are conservative in that the effective body temperatures of cartilaginous fishes are underestimated and those of reptiles are overestimated. Inserting the true values of mean body temperatures would thus strengthen the link with brain size.

These findings further clarify endothermy’s role in brain size. Ectothermic cartilaginous fishes show fundamentally the same relationship between adult body size, newborn size, and adult brain size as endothermic precocial birds, which also lack posthatching provisioning (Figs. 1 and 3*C*). However, posthatching provisioning is rare in ectotherms (41), and the difference between altricial and precocial birds in brain size (18, 42) shows that marked encephalization requires provisioning both before and after hatching. Endothermy provides this combination (Fig. 4*C*). High posthatching parental association and therefore provisioning is made possible by the high level of sustained activity due to aerobic metabolism (54), as argued previously (55, 56). But endothermy also facilitates greater average prehatching provisioning, i.e., incubation, as shown by the contrast in offspring sizes between precocial (endothermic) birds and (ectothermic) reptiles, both of which are amniotic, terrestrial, and lacking posthatching provisioning (*SI Appendix,* Fig. S1). This second effect presumably reflects the greater safety of the developing eggs and the higher, more stable body temperatures due to incubation that accelerate development (19). Overall, endothermy permitted the evolution of larger relative brain size of birds and mammals by increasing both pre- and posthatching provisioning.

Nonetheless, endothermy probably did not evolve for this function [even if this is sometimes claimed: (55, 56)]. In birds, precociality is the ancestral state (18, 42), which thus did not involve posthatching provisioning. Instead, endothermy may have evolved to enable sustained flight (54). Likewise, in mammals, endothermy may have evolved to enable continuous activity during the night when basking is impossible, and posthatching provisioning was only one of several accompanying benefits (57). Endothermy thus served as a preadaptation or exaptation for encephalization.

The conclusions of comparative studies are inevitably limited by the quality and quantity of data. First, the paucity and intersource variability of brain size data for ectotherms may have reduced statistical significance. However, the general trends were generally the same across all classes. Second, there were not enough brain size data for the few species of fishes or amphibians with postnatal provisioning to assess the latter’s effect on brain size in ectotherms. However, although future work should assess these effects, any effects on brain size of postnatal provisioning will inevitably be additive to the ones due to prenatal provisioning. Third, more data on body temperatures and their fluctuations in the ectotherm classes would be needed to definitively differentiate between the role of body temperature (both mean and variance) and metabolic rates on brain size. However, both alternatives are consistent with the expensive brain framework examined here. In general, future work should refine the explanation of the effect of body temperature on brain size.

We conclude that the broad taxonomic distribution of vertebrate brain sizes is greatly affected by the high energy costs of brain development and maintenance. Encephalization could only take off once parents were able to invest more into individual offspring and could sustain higher body temperatures (Fig. 4*C*). These independent evolutionary innovations allowed organisms to benefit from the cognitive and sensorimotor benefits supported by larger brains, and initiated coevolutionary processes that paved the way for the emergence of hyperencephalized lineages, most conspicuously our own.

## Materials and Methods

### Data Collection.

#### Brain data.

Brain size data were obtained from several sources. For fishes we used FishBase (58), supplemented for cartilaginous fishes with data from ref. 59. In all cases, we removed data on immature animals wherever possible as well as various obvious errors. First, species were removed if their relative brain size was a highly significant outlier, defined as being less than 0.8 or greater than 1.2 times the expected value of the log brain on log body regression of specimens within a species. Second, if there were less than five specimens within a species, species were removed if their relative brain size was less than 0.6 or greater than 1.7 times the expected value based on a regression of log brain on log body within a genus. For amphibians and reptiles, we used (15), supplemented for turtles and crocodiles with data from ref. 60 and additional sources detailed in the supplementary data file. For birds we used the data from ref. 18 and for mammals mainly from ref. 61 as well as additional sources (*SI Appendix*).

#### Newborn sizes.

The hatchling lengths of cartilaginous fishes were gathered from FishBase (58) and additional resources located via Google scholar, as listed in the supplementary data (as for all following cases sourced this way). The length-weight equation from ref. 58 was applied to convert these lengths into body mass in grams. For ray-finned fishes, egg diameters (in cm) were sourced from ref. 58 and additional resources, through systematic species-specific searches conducted in Google scholar. These diameters were then converted to cubic centimeters (cm^3^) and directly converted to weights, assuming a density of 1. Egg diameters of amphibians, measured in centimeters, were taken from multiple sources (*SI Appendix*). These diameters were similarly converted to mass. Hatchling length of squamate reptiles were collected from ref. 62 and additional resources. The length-weight formula from ref. 62 was then applied to convert these lengths into body mass. Egg masses for turtles and crocodiles were collected via Google scholar. Because eggshells are thin and data are sparse, we did not convert them into newborn sizes. Finally, hatchling mass data for birds were primarily derived from ref. 63 and supplemented with egg mass data obtained from ref. 18. To enable comparison with newborn sizes in other lineages, we derived a conversion formula to estimate hatchling mass from egg mass (n = 453, slope = 0.936, intercept = −0.158, R^2^ = 0.970), by regressing log-transformed egg mass against log-transformed hatchling mass. Neonate mass data on mammals were taken from multiple sources (*SI Appendix*), with the mean taken when multiple measurements were available. Note that the absence of hatchling masses forced us to use egg masses for ray-finned fishes and amphibians, but this modest difference led us to overestimate their newborn sizes, and is therefore conservative, given the small sizes of most eggs in these classes.

#### Egg care behaviors.

*SI Appendix,* Table S1 contains the definitions of egg care behaviors by lineage. The main sources for egg care behavior were as follows: for cartilaginous fishes (59), for ray-finned fishes (58), for amphibians (64), and for squamate reptiles (65). In all cases, we searched in Google scholar for data on species not in the sources listed above. We categorized four megapode species in birds as egg abandoning since they do not incubate their eggs. All mammals were categorized as provisioning newborns.

#### Body temperature.

We collected body temperature data across all vertebrate groups as follows. For amphibians and reptiles, we collected body temperature data for wild animals that were active, thus excluding laboratory records of preferred body temperature or data from brumating wild animals [primary source (15)]. We used the median of the reported minimum and maximum body temperature values, which provides an estimate of average body temperature during normal daily activity. In total, we compiled mean body temperature data for 41 amphibian species and 112 reptile species. For birds and mammals, we collected body temperature data when the animals were resting and normothermic. The main source for body temperature data for birds was (66), and for mammals (67). Because in most fishes, body temperature is closely tied to water temperature, we used the modal water temperatures for fishes from FishBase (58) as a proxy for body temperature. Note that this procedure is conservative, especially for cartilaginous fishes (*Discussion*).

#### Ambient temperature.

We collected ambient temperature data across the geographic distribution of each species. For mammals, mean ambient temperatures within species’ distribution ranges were obtained from ref. 68 and supplemented by ref. 67. For birds, ambient temperature data were sourced from ref. 69.

#### BMRs.

BMR values for mammals were compiled from multiple sources [primarily (67)], for birds from various sources (details provided in the Supplementary Data). Since BMR is strongly dependent on body size, we averaged BMR values only for records with body mass within ±20% of the species-specific mean, to reduce within-species variability. To control for body size, we calculated the residuals of BMR relative to body mass within each class. For ectotherms, insufficient data were found for analysis.

### Statistical Analyses.

Because hagfish, lampreys, lungfishes, and coelacanths have only a few extant members, most analyses could only be done for cartilaginous fishes, ray-finned fishes, amphibians, reptiles, birds, and mammals (*SI Appendix,* Table S2).

To account for phylogenetic relationships within a class, we built a multitree phylogeny based on recently published, time-calibrated trees for cartilaginous fishes (70), reptiles (71), and amphibians (72). For each lineage, we randomly selected 100 trees to generate Maximum Clade Credibility (MCC) trees using TreeAnnotator (73). The phylogeny for birds was obtained from the most comprehensive, up to date MCC phylogenetic tree [Aves 1.5, (74)]. A consensus mammal phylogenetic tree was obtained from ref. 61 and for ray-finned fishes from ref. 75.

#### Within classes.

To establish correlated evolution between newborn size and brain size, we first conducted analyses within classes rather than in the total sample. We used this approach to avoid intractable interaction effects due to the presence of potentially important variables that are universal or nearly so in one lineage but absent in another (e.g., aquatic life, flight, number of limbs, metamorphosis, endothermy), yet could independently affect brain size.

We first fitted phylogenetically controlled mixed models in the R package MCMCglmm (76) to evaluate the impact of egg care behaviors on newborn mass in cartilaginous fishes, ray-finned fishes, amphibians, and reptiles. To control for the allometric relationship between newborn mass and body mass, we applied a log10-transformation to both variables and included adult body mass as a fixed variable alongside egg care behavior.

We next investigated the impact of different forms of egg care and prehatching provisioning on newborn size and brain size evolution in each class. In cartilaginous fishes, we examined the extent of correlated evolution between newborn mass and brain mass among egg abandoners, egg bearers, and offspring provisioners. In ray-finned fishes, amphibians, and reptiles, our analysis focused on the correlation between newborn mass and brain mass among egg abandoners, egg guarders, and egg bearers. For birds, we assessed the correlated evolution between newborn mass and brain mass across all species after removing 4 megapode species. For mammals, since all species provision their embryos before birth, we assessed the correlated evolution between newborn mass and brain mass across all species. In all analyses, all variables were log10-transformed, and body mass was included as a fixed variable to account for allometric effects.

We further tested the effect of body temperature and newborn size on brain size within classes with adequate sample sizes. All variables were log10-transformed, and body mass was included as a fixed variable.

#### Among classes.

We examined the relationship between brain size and both body temperature and newborn size across all vertebrate combined. This required a phylogenetically controlled mixed model among brain and newborn size on body size among all vertebrates.

To do so, we created a phylogeny that included all vertebrates, as follows. The individual phylogenetic subsets representing the lampreys, cartilaginous fishes, ray-finned fishes, amphibians, reptiles, birds, and mammals were combined with connecting branches connecting the Last Common Ancestors (LCAs). Using the mean of node ages from the LCAs inferred by ref. 77, we first connected Aves and crocodilians at the LCA node dated 244.8 million years ago (Myr). This was then connected with testudomorphs at the LCA node set at 261.4 Myr. Next, we connected this with lepidosaurs at the LCA node set at 279.9 Myr to form a sauropsid phylogenetic tree. This sauropsid tree was then connected with the Mammalia tree at the LCA node at 318.9 Myr to form the Amniota tree. This Amniota phylogenetic tree was further connected with amphibians at the LCA node set at 351.7 Myr to form a Tetrapoda phylogenetic tree. The latter was connected to the tree of Actinopterygii at the LCA node set at 429.0 Myr. This Osteichthyes tree was further connected with the Chondrichthyes tree at the LCA node set at 462.4 Myr, and finally connected to the agnathan phylogenetic tree at the LCA node set at 563.4 Myr.

We built a phylogenetically controlled mixed model in MCMCglmm (76) including all vertebrates to test the effect of newborn mass (log10-transformed) and body temperature and their interaction on brain mass (log10-transformed), with body mass (log10-transformed) included as a fixed effect.

#### Phylogenetic path analyses.

To test the alternative hypothesis that metabolic rates rather than body temperature affected brain size, we conducted phylogenetic path analyses, which explored potential direct and indirect relationships among six traits: ambient temperature (Ta), body temperature (Tb), BMR, newborn size, adult body mass, and brain size. Birds (n = 177) and mammals (n = 240) were analyzed separately using the phylopath package (78) in R, which performs phylogenetic path analysis based on phylogenetic generalized least squares (PGLS), thereby accounting for shared evolutionary history. Due to the extremely high collinearity between BMR and body mass (R^2^ > 0.94 in both groups), and because the specimens used for BMR measurements often differed in body mass from those in the brain size dataset, we used the residuals of BMR, brain size, and newborn size after regressing each trait against body mass in the dataset of origin.

We constructed 10 candidate path models that in several key causal assumptions, including whether BMR influences Tb, and which combination of Ta, Tb, and BMR have direct effects on brain size (*SI Appendix*, Fig. S2). For each model, the implied conditional independencies (d-separation statements) were evaluated using Fisher’s C-statistic. Models that passed the C test (*P* > 0.05) were considered consistent with the data and were then ranked using the CICc (C-statistic Information Criterion corrected for small sample size). Models with ΔCICc < 2 were considered equivalently supported and were averaged using their CICc weights.

#### General procedures.

All data were scaled and centered before all analyses. All models were fitted using an MCMC-based mixed modeling approach using the package MCMCglmm (76). We used chains of 500,000 iterations, with the first 100,000 iterations discarded as burn-ins and thinned every 40 iterations. Visual inspection of the final MCMC samples did not show any sign of autocorrelation. For all models, inverse-gamma priors were used for residual variances (parameterized as inverse-Wishart with V = 1 and ν = 0.002). The prior for phylogenetic effect was formed as a weakly informative half-Cauchy density (parameter expanded priors with V = 1, alpha.mu = 0 and alpha.V = 100,000). We implemented all statistical analyses in R 4.1.1 (79).

## Supplementary Material

Appendix 01 (PDF)

## Data Availability

The data and R code has been deposited in the Dryad Digital Repository (80).
