# Supplementary material for: Parental investment and body temperature explain encephalization in vertebrates
Source: Proc Natl Acad Sci U S A. 2025 Nov 3;122(45):e2506145122. doi: 10.1073/pnas.2506145122 (PMC12625846; doi:10.1073/pnas.2506145122)
Supplement: Supplementary file 1 — Appendix 01 (PDF) [file pnas.2506145122.sapp.pdf]

Supporting Information for

Parental investment and body temperature explain encephalization in vertebrates

Authors: Zitan Song*, Michael Griesser & Carel P. van Schaik

*Corresponding author: Zitan Song

Email: songzitan@gmail.com

This PDF file includes:

Figures S1 to S4
Tables S1 to S16
SI References

Figure S1.

A **B**

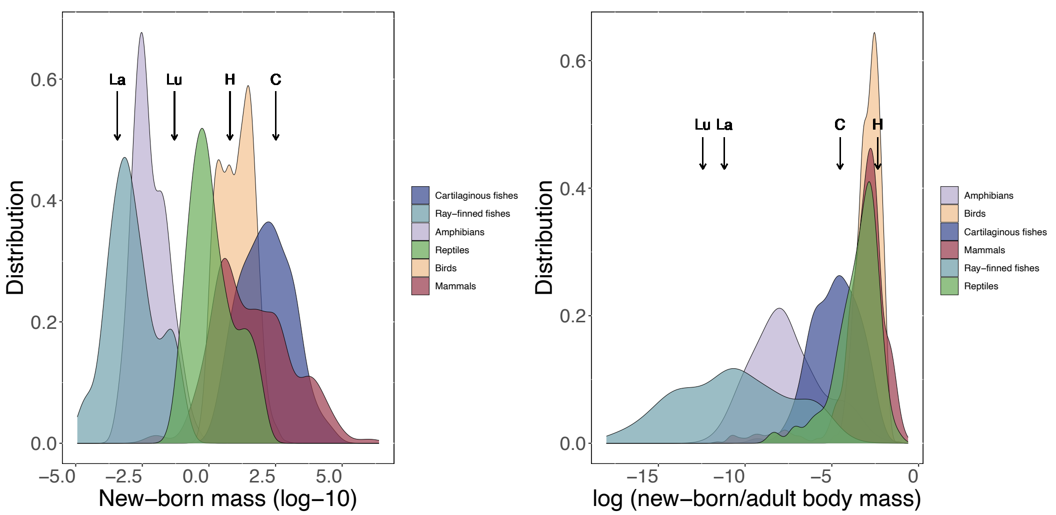

Fig. S1. Size distributions of newborns for each class of vertebrates included in this study. (**A**) absolute mass of hatchlings or newborns. Note the gap at around newborn size 0.1 gram (or an egg size approximately 6 mm diameter). (**B**) The distribution of the (log-transformed) ratio of newborn mass to adult mass for the classes in this study. The arrows indicate mean sizes of small classes: H = hagfishes; La = lampreys; C = coelacanths; and Lu = lungfishes.

Figure S2.

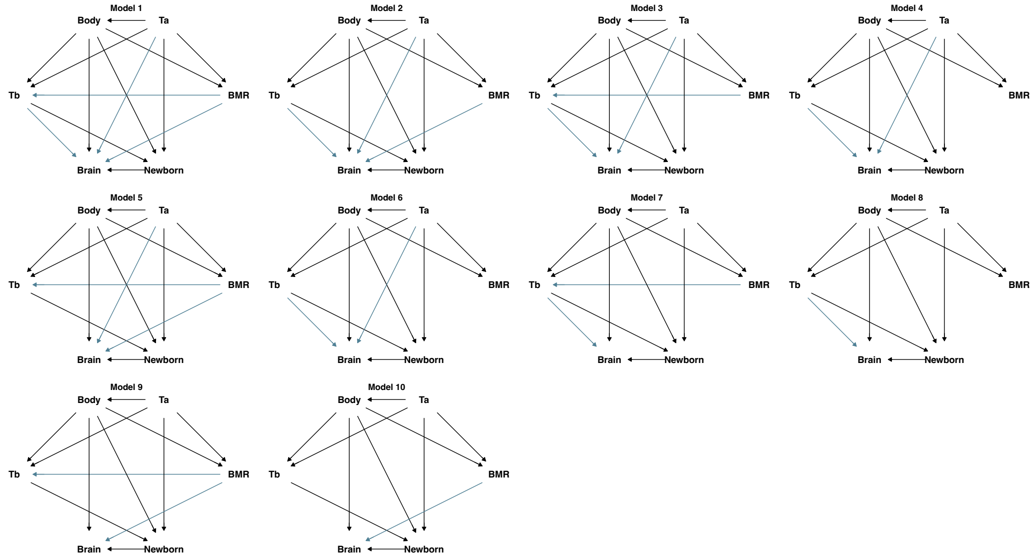

Fig. S2. Ten candidate path models were tested using phylogenetic path analysis to explore the relationships among body size (Body), ambient temperature (Ta), body temperature (Tb), residual basal metabolic rate (BMR), residual newborn size (Newborn), and residual brain size (Brain) in birds and mammals. Models were evaluated based on d-separation tests, with overall fit quantified using Fisher's C statistic and ranked using CICc.

Figure S3.

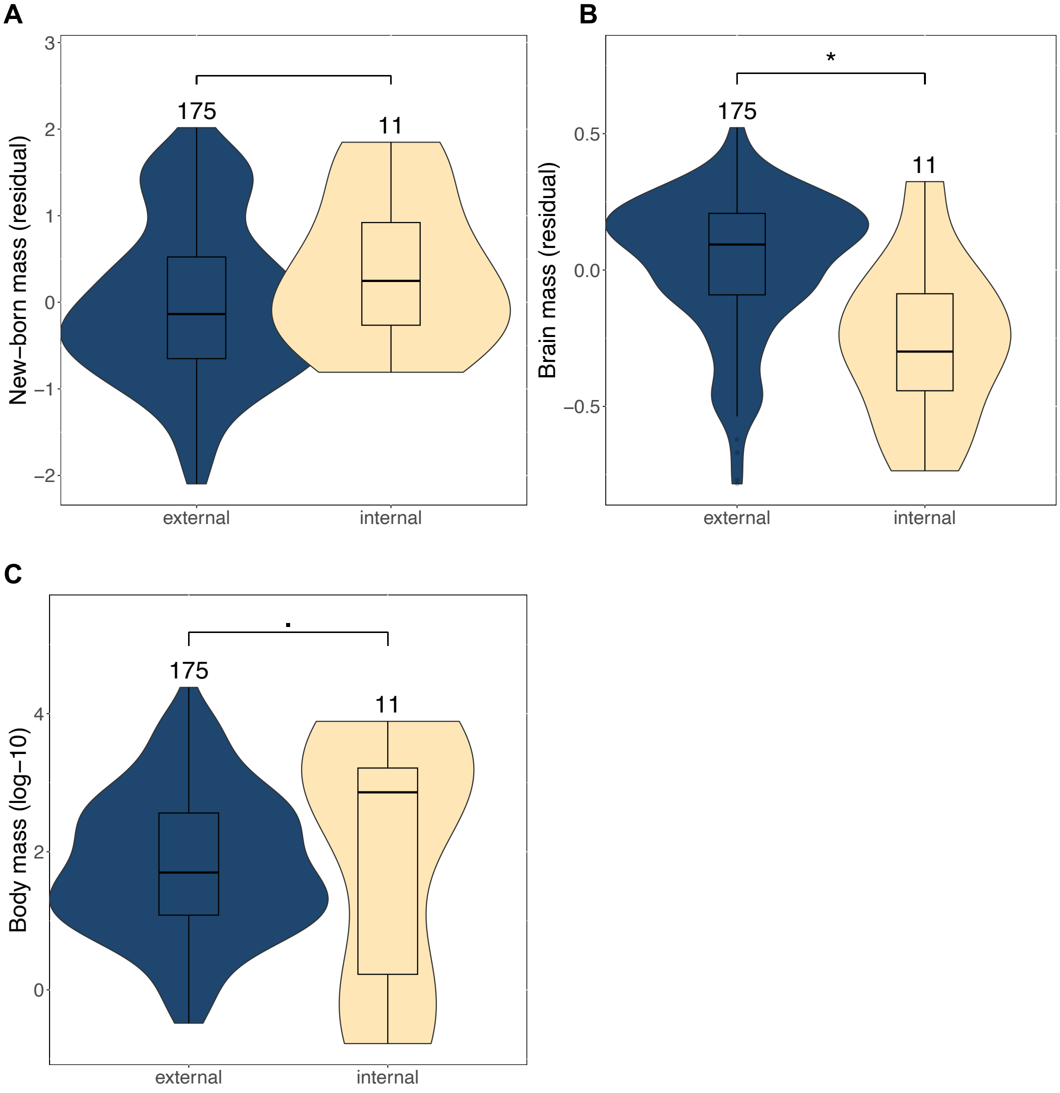

Fig. S3. Effects of fertilization mode in ray-finned fishes on various attributes. **(A-C)** Comparisons of relative newborn mass (**A**), relative adult brain mass (**B**) and adult body mass (**C**) in relation to the internal or external fertilization in ray-finned fishes. The number of species is indicated above each violin plots. For statistics see Table S16.

Figure S4.

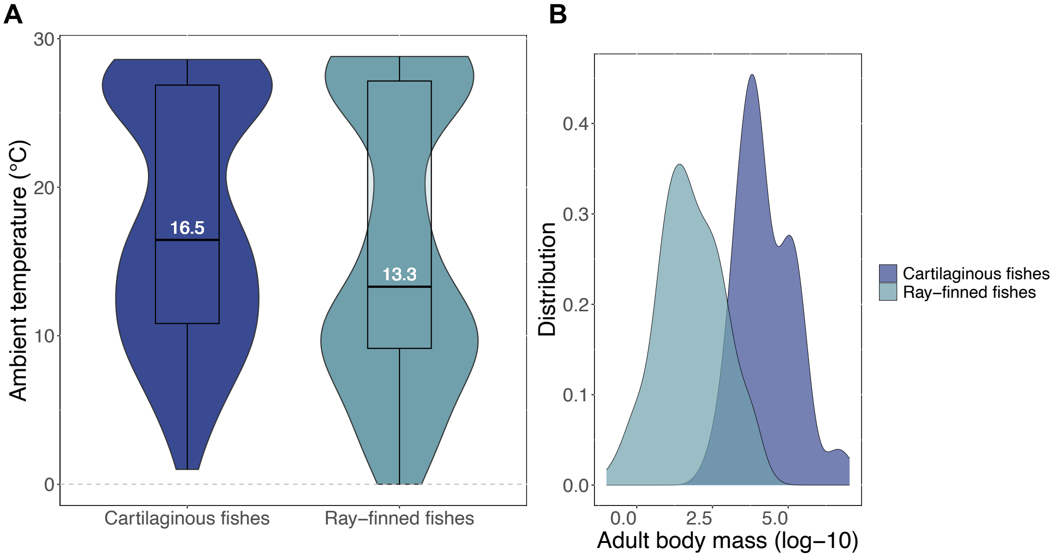

Fig. S4. The difference between cartilaginous fishes and ray-finned fishes in body size and modal water temperature. **(A)** Distribution of species means of water temperatures. **(B)** Size distribution of species values of adult body mass in the samples available in this study (using Gaussian fit).

Table S1. Criteria used to place species into the various categories of egg care, by lineage. Definitions for cartilaginous fishes follow (1), for ray-finned fishes follow (2), for amphibians follow (3), for squamate reptiles follow (4), and for birds follow (5).

Lineage	Egg care behaviour	Egg care behaviour categories	Sample size
Cartilaginous fishes	**Egg-laying**: offspring develops in a leathery egg case, nourishment is provided by the yolk sac	Abandon	26
	**Yolk-only viviparity**: offspring develops inside the mother, nourishment is provided by the yolk sac	Bear	36
	**Live-bearing with uterine milk**: offspring develops inside the mother, nourishment is provided by the yolk sac and 'uterine milk' secreted by the mother;<br>**Placental viviparity**: offspring develops inside the mother, nourishment is filtered from the circulatory system of the mother via a placental connection;<br>**Live-bearing with oophagy**: offspring develops inside the mother, nourishment is provided by the yolk sac, and after the yolk is fully absorbed embryos feed on unfertilised eggs	Pre-hatching provisioning	58
Ray-finned fishes	**Brood hiders**: eggs are deposited in inconspicuous places;<br>**Open water/substratum egg scatterers**: eggs are left after spawning in the water column or on any substrate	Abandon	107
	**Clutch tenders**: eggs are guarded at the water surface, on underside of objects or any substrate<br>**Nesters**: eggs are deposited and guarded in nests	Guard	42
	**External brooders**: eggs are incubated externally on parental body;<br>**Internal live bearers**: internal fertilization of eggs, with development taking place inside the maternal body	Bear	47
Amphibians	**Abandon**: eggs are left after spawning in the water column or placed on any substrate	Abandon	90
	**Egg attendance**: parent(s) remaining, full- or part-time, with the eggs at a fixed location	Guard	25
	**Egg brooding**: eggs are carried on the parent's body (e.g., on the back or between the hindlegs) or within it (e.g., vocal sacs, stomach), ending with the release of tadpoles (no direct development) or froglets (direct development);<br>**Live bearing**: female gestation of offspring in the oviducts from fertilisation to birth	Bear	4
Reptiles	**Egg laying**	Abandon	77
	**Egg Attendance (Egg manipulation and/or nest defense);**<br>**Parent–offspring associations**: parent–offspring associations that persist beyond the birthing/hatching period)	Guard	24
	**Viviparity and incubation**	Bear	34
Birds	**Megapode**: parents bury eggs but do not incubate them (using heat from external sources)	Abandon	4

	**Precocial**: young hatch with open eyes and leave the nest within 2 days after hatching	Pre-hatching provisioning	299
	**Semi-precocial**: young hatch with open eyes and are soon capable of walking, but they remain in the nest to be fed by parents	Pre-&Post-hatching provisioning	126
	**Semi-altricial**: young hatch with open eyes and are feathered, but remain in the nest to be fed by their parents		144
	**Altricial**: young hatch with closed eyes and largely lack feathers, and are fed by parents in the nest		563
Mammals	**Lactation**	Pre-&Post-hatching provisioning	855

Table S2. Classes of vertebrates included in the analysis, with their popular names and sample sizes.

Class	Popular name	N of species in sample (with mass of adult brain and body and mass of newborn)	N of species with care category	N of species with body temperature
Myxini	Hagfishes	2	2	2
Hyperoartia	Lampreys	14	14	4
Chondrichthyes	Cartilaginous fishes	120	120	118
Actinopterygii	Ray-finned fishes	210	196	159
Actinistia	Coelacanths	1	1	1
Dipnoi	Lungfishes	2	2	0
Amphibia	Amphibians	130	119	41
Reptilia	Reptiles	190	135	112
Aves	Birds	1136	1136	348
Mammalia	Mammals	855	855	274

Table S3. Phylogenetically controlled mixed model in the R package *MCMCglmm* assessing the effect of egg care on newborn mass (log-10) in the classes.

	Posterior Mean	lower ▪ upper 95% CI	effective sample size	p MCMC
		**Cartilaginous fishes**		
**Egg abandoning vs live bearing and pre-hatching provisioning**				
Intercept	-2.717	-4.306 ▪ -1.088	10000	0.001
**Body (log-10)**	**1.426**	**1.224 ▪ 1.633**	**10000**	**<0.001*****
Egg care (*egg abandoning vs live bearing*)	0.808	-0.194 ▪ 1.917	10344	0.129
**Egg care (*egg abandoning vs pre-hatching provisioning*)**	**1.730**	**0.627 ▪ 2.781**	**10000**	**0.003****
**Live bearing vs pre-hatching provisioning**				
Intercept	-0.598	-1.542 ▪ 0.385	9753	0.223
**Body (log-10)**	**0.610**	**0.511 ▪ 0.708**	**8816**	**<0.001*****
Egg care (*live bearing vs pre-hatching provisioning*)	0.383	-0.062 ▪ 0.810	10000	0.081
		**Ray-finned fishes**		
**Egg abandoning vs egg guarding and live bearing**				
Intercept	-2.737	-4.100 ▪ -1.309	10000	<0.001
**Body (log-10)**	**0.171**	**0.064 ▪ 0.272**	**11010**	**<0.001*****
Egg care (*egg abandoning vs egg guarding*)	0.197	-0.155 ▪ 0.558	9703	0.273
**Egg care (*egg abandoning vs live bearing*)**	**0.800**	**0.347 ▪ 1.272**	**10000**	**0.001****
**Egg guarding and live bearing**				
Intercept	-2.980	-5.306 ▪ -0.556	10000	0.017
**Body (log-10)**	**0.222**	**0.053 ▪ 0.385**	**10000**	**0.001****
**Egg care (*egg guarding vs live bearing*)**	**0.679**	**0.138 ▪ 1.302**	**10000**	**0.023***
		**Amphibians**		
**Egg abandoning vs egg guarding and live bearing**				
Intercept	-2.547	-3.184 ▪ -1.879	10000	<0.001
**Body (log-10)**	**0.366**	**0.199 ▪ 0.527**	**10297**	**<0.001*****
**Egg care (*egg abandoning vs egg guarding*)**	**0.412**	**0.085 ▪ 0.725**	**9473**	**0.012***
Egg care (*egg abandoning vs live bearing*)	0.577	0.004 ▪ 1.195	9065	0.053
**Egg guarding and live bearing**				

Intercept	-1.989	-2.671 ▪ -1.323	10000	<0.001
**Body (log-10)**	**0.341**	**0.049 ▪ 0.619**	**10000**	**0.022***
Egg care (*egg guarding vs live bearing*)	-0.090	-0.815 ▪ 0.637	9833	0.784
		**Reptiles**		
**Egg abandoning vs egg guarding and live bearing**				
Intercept	-0.999	-1.414 ▪ -0.598	10000	<0.001
**Body (log-10)**	**0.617**	**0.543 ▪ 0.683**	**10000**	**<0.001*****
Egg care (*egg abandoning vs egg guarding*)	0.103	-0.048 ▪ 0.261	9671	0.198
Egg care (*egg abandoning vs live bearing*)	0.015	-0.144 ▪ 0.177	10000	0.844
**Egg guarding and live bearing**				
Intercept	-1.209	-1.666 ▪ -0.760	10000	<0.001
**Body (log-10)**	**0.722**	**0.618 ▪ 0.818**	**10000**	**<0.001*****
Egg care (*egg guarding vs live bearing*)	-0.046	-0.265 ▪ 0.153	10000	0.660

Significant predictors are highlighted in bold.

Table S4. Phylogenetically controlled mixed model in the R package *MCMCglmm* assessing the effect of relative newborn mass on relative adult brain mass for various egg care categories.

	Posterior Mean	lower ▪ upper 95% CI	effective sample size	p MCMC
		**Cartilaginous fishes**		
**Egg abandoning (n = 26)**				
Intercept	0.146	-0.714 ▪ 1.099	10499	0.708
**Body (log-10)**	**0.820**	**0.562 ▪ 1.083**	**10000**	**<0.001*** **
Newborn mass (log-10)	-0.099	-0.346 ▪ 0.143	9468	0.412
**Live bearing (n = 36)**				
Intercept	0.010	-1.080 ▪ 1.185	10000	0.990
**Body mass (log-10)**	**0.672**	**0.458 ▪ 0.886**	**10000**	**<0.001*** **
Newborn mass (log-10)	0.120	-0.076 ▪ 0.315	9720	0.222
**Pre-hatching provisioning (n = 58)**				
Intercept	-0.387	-1.382 ▪ 0.595	10000	0.431
**Body mass (log-10)**	**0.678**	**0.560 ▪ 0.790**	**10000**	**<0.001*** **
**Newborn mass (log-10)**	**0.278**	**0.120 ▪ 0.423**	**10000**	**<0.001*** **
		**Ray-finned fishes**		
**Egg abandoning (n = 107)**				
Intercept	-0.333	-0.991 ▪ 0.390	10000	0.337
**Body mass (log-10)**	**0.824**	**0.761 ▪ 0.888**	**10000**	**<0.001*** **
Newborn mass (log-10)	0.009	-0.070 ▪ 0.090	10000	0.831
**Egg guarding (n = 42)**				
Intercept	0.414	-0.718 ▪ 1.594	10000	0.468
**Body mass (log-10)**	**0.887**	**0.756 ▪ 1.023**	**10000**	**<0.001*** **
Newborn mass (log-10)	-0.015	-0.164 ▪ 0.136	10000	0.834
**Live bearing (n = 47)**				
Intercept	0.004	-1.198 ▪ 1.311	10302	0.992
**Body mass (log-10)**	**0.619**	**0.520 ▪ 0.715**	**10000**	**<0.001*** **
**Newborn mass (log-10)**	**0.134**	**0.009 ▪ 0.255**	**10000**	**0.034***
		**Amphibians**		
**Egg abandoning (n = 90)**				
Intercept	-0.287	-0.820 ▪ 0.210	10000	0.225
**Body mass (log-10)**	**0.780**	**0.659 ▪ 0.898**	**10000**	**<0.001*** **
Newborn mass (log-10)	0.000	-0.120 ▪ 0.116	10000	0.998
**Egg guarding (n = 25)**				

Intercept	0.136	-0.434 ▪ 0.796	10000	0.619
**Body mass (log-10)**	**0.710**	**0.458 ▪ 0.936**	**10000**	**<0.001*****
**Newborn mass (log-10)**	**0.265**	**0.029 ▪ 0.494**	**9942**	**0.028***
		**Reptiles**		
**Egg abandoning (n = 77)**				
Intercept	-0.033	-0.450 ▪ 0.387	10274	0.867
**Body mass (log-10)**	**1.059**	**0.910 ▪ 1.210**	**10000**	**<0.001*****
Newborn mass (log-10)	-0.031	-0.190 ▪ 0.130	10000	0.698
**Egg guarding (n = 24)**				
Intercept	0.080	-0.236 ▪ 0.395	10000	0.599
**Body mass (log-10)**	**0.451**	**0.172 ▪ 0.719**	**9442**	**0.001****
**Newborn mass (log-10)**	**0.460**	**0.199 ▪ 0.713**	**9496**	**0.002****
**Live bearing (n = 34)**				
Intercept	-0.009	-0.606 ▪ 0.606	10000	0.969
**Body mass (log-10)**	**0.925**	**0.716 ▪ 1.129**	**9356**	**<0.001*****
Newborn mass (log-10)	0.081	-0.134 ▪ 0.296	9549	0.437
		**Birds**		
**Incubating birds (n = 1132)**				
Intercept	-0.382	-0.620 ▪ -0.142	10000	0.003
**Body mass (log-10)**	**0.724**	**0.673 ▪ 0.779**	**10000**	**<0.001*****
**Newborn mass (log-10)**	**0.345**	**0.286 ▪ 0.402**	**10000**	**<0.001*****
		**Mammals**		
**All mammals (n = 855, similar model to Table 1)**				
Intercept	0.178	-0.116 ▪ 0.475	9661	0.240
**Body mass (log-10)**	**0.576**	**0.541 ▪ 0.612**	**10000**	**<0.001*****
**Newborn mass (log-10)**	**0.286**	**0.245 ▪ 0.330**	**10000**	**<0.001*****

Significant predictors are highlighted in bold.

Table S5. Slopes and intercepts for allometric relationships between adult brain mass and newborn mass per class, built using phylogenetic generalized least squares models in the R package *phylolm*.

Class	Parameters	estimate	se	t value	p value
**Lampreys**	Intercept	-2.102	1.111	-1.891	0.083
**(n = 14)**	Newborn mass (log-10)	**-0.076**	0.322	-0.236	0.818
				lambda = 0.000, r2 = 0.005	
**Cartilaginous fishes**	Intercept	-0.027	0.229	-0.117	0.907
**(n = 120)**	Newborn mass (log-10)	**0.533**	0.038	14.048	<0.001***
				lambda = 0.925, r2 = 0.626	
**Ray-finned fishes**	Intercept	-0.251	0.541	-0.463	0.644
**(n =210)**	Newborn mass (log-10)	**0.161**	0.050	3.243	0.001**
				lambda = 0.980, r2 = 0.048	
**Amphibians**	Intercept	-1.043	0.295	-3.530	<0.001
**(n = 130)**	Newborn mass (log-10)	**0.233**	0.056	4.145	<0.001***
				lambda = 0.943, r2 = 0.118	
**Reptiles**	Intercept	-0.725	0.182	-3.980	<0.001
**(n = 190)**	Newborn mass (log-10)	**0.669**	0.036	18.549	<0.001***
				lambda = 0.925, r2 = 0.647	
**Birds**	Intercept	-0.608	0.079	-7.654	<0.001
**(n =1136)**	Newborn mass (log-10)	**0.804**	0.013	64.156	<0.001***
				lambda = 0.946, r2 = 0.784	
**Mammals**	Intercept	0.723	0.257	2.813	0.005
**(n =855)**	Newborn mass (log-10)	**0.653**	0.014	46.950	<0.001***
				lambda = 0.972, R^2 = 0.721	

Note: Due to our conservative rule for assigning adulthood, some specimens, mostly ectotherms (400 out of 2661), were classified as subadult. In these cases, adult brain mass was estimated by predicting brain mass based on adult body mass, and then adding the residual brain mass from the model for each class to retain species-specific trends in brain mass relative to body mass (note that adult brain mass was used here solely for illustrative purposes and was not included in other analyses). The slope and intercept were calculated based on adult brain mass and newborn mass.

Table S6. Slope and intercept for allometric relationships between adult brain size and body size for the various classes, built using phylogenetic generalized least squares models in the R package *phylolm*.

Class	Parameters	estimate	se	t value	p value
**Lamprey**	Intercept	-2.366	0.138	-17.171	<0.001
**(n = 14)**	Body mass (log-10)	**0.355**	0.043	8.237	<0.001***
			lambda = 0.908, r2 = 0.850		
**Cartilaginous fishes**	Intercept	-0.929	0.190	-4.882	<0.001
**(n = 120)**	Body mass (log-10)	**0.424**	0.023	18.222	<0.001***
			lambda = 0.958, r2 = 0.738		
**Ray-finned fishes**	Intercept	-1.816	0.197	-9.197	<0.001
**(n =210)**	Body mass (log-10)	**0.514**	0.014	36.583	<0.001***
			lambda = 0.975, r2 = 0.865		
**Amphibians**	Intercept	-2.126	0.116	-18.324	<0.001
**(n = 130)**	Body mass (log-10)	**0.523**	0.033	16.081	<0.001***
			lambda = 0.665, r2 = 0.669		
**Reptiles**	Intercept	-1.826	0.106	-17.287	<0.001
**(n = 190)**	Body mass (log-10)	**0.569**	0.015	37.728	<0.001***
			lambda = 0.887, r2 = 0.883		
**Birds**	Intercept	-1.097	0.061	-18.031	<0.001
**(n =1136)**	Body mass (log-10)	**0.588**	0.007	84.957	<0.001***
			lambda = 0.918, r2 = 0.864		
**Mammals**	Intercept	-0.852	0.174	-4.901	<0.001
**(n =855)**	Body mass (log-10)	**0.582**	0.008	72.961	<0.001***
			lambda = 0.968, R^2 = 0.862		

Table S7. Phylogenetically controlled mixed model in the R package *MCMCglmm* assessing the effect of temperature and newborn mass on brain mass within classes.

	Posterior Mean	lower ▪ upper 95% CI	effective sample size	p MCMC
	**Cartilaginous fishes (n = 118)**			
Intercept	-0.066	-0.677 ▪ 0.544	10000	0.843
**Body (log-10)**	**0.617**	**0.539 ▪ 0.694**	**9566**	**<0.001*****
**Newborn mass (log-10)**	**0.200**	**0.096 ▪ 0.305**	**10000**	**<0.001*****
**Body temperature**	**0.180**	**0.078 ▪ 0.279**	**10624**	**<0.001*****
	**Ray-finned fishes (n = 159)**			
Intercept	-0.036	-0.789 ▪ 0.760	10367	0.929
**Body (log-10)**	**0.761**	**0.709 ▪ 0.809**	**10000**	**<0.001*****
Newborn mass (log-10)	0.012	-0.044 ▪ 0.067	8488	0.681
Body temperature	0.051	-0.011 ▪ 0.116	10000	0.114
	**Amphibians (n = 41)**			
Intercept	0.047	-0.380 ▪ 0.489	10000	0.799
**Body (log-10)**	**0.824**	**0.644 ▪ 1.004**	**9417**	**<0.001*****
Newborn mass (log-10)	0.103	-0.089 ▪ 0.304	10000	0.296
Body temperature	0.046	-0.157 ▪ 0.248	9366	0.651
	**Reptiles (n = 112)**			
Intercept	0.062	-0.232 ▪ 0.383	10000	0.682
**Body (log-10)**	**0.919**	**0.783 ▪ 1.052**	**8639**	**<0.001*****
Newborn mass (log-10)	0.049	-0.068 ▪ 0.167	8679	0.414
Body temperature	0.044	-0.013 ▪ 0.101	10625	0.135
	**Birds (n = 348)**			
Intercept	-0.366	-0.629 ▪ -0.108	10000	0.0070
**Body (log-10)**	**0.762**	**0.669 ▪ 0.861**	**10000**	**<0.001*****
**Newborn mass (log-10)**	**0.343**	**0.234 ▪ 0.447**	**10000**	**<0.001*****
**Body temperature**	**0.032**	**0.010 ▪ 0.054**	**10000**	**0.008****
	**Mammals (n = 274)**			
Intercept	0.337	-0.028 ▪ 0.689	10000	0.062
**Body (log-10)**	**0.649**	**0.593 ▪ 0.707**	**9960**	**<0.001*****
**Newborn mass (log-10)**	**0.292**	**0.226 ▪ 0.365**	**9796**	**<0.001*****
**Body temperature**	**0.027**	**0.010 ▪ 0.045**	**10000**	**0.006****

Significant predictors are highlighted in bold.

Table S8. Model selection results from phylogenetic path analysis. Each candidate model was evaluated using Fisher's C test and ranked by the C-statistic Information Criterion corrected for small sample size (CICc). Model structures are shown in Figure S2.

Models	k	q	Fisher's C	p	CICc	Delta CICc	weight
			**Birds (n = 177)**				
**m1**	**1**	**20**	**3.249**	**0.197**	**48.633**	**0.000**	**0.444**
**m3**	**2**	**19**	**6.275**	**0.180**	**49.116**	**0.483**	**0.349**
m2	2	19	9.668	0.046	52.509	3.875	0.064
m4	3	18	12.694	0.048	53.024	4.390	0.049
m6	3	18	12.694	0.048	53.024	4.390	0.049
m5	2	19	11.300	0.023	54.141	5.507	0.028
m7	3	18	15.669	0.016	55.998	7.364	0.011
m9	3	18	18.972	0.004	59.301	10.668	0.002
m8	4	17	22.088	0.005	59.937	11.304	0.002
m10	4	17	25.392	0.001	63.241	14.607	0.000
			**Mammals (n = 240)**				
**m7**	**3**	**18**	**2.709**	**0.844**	**41.804**	**0.000**	**1.000**
m3	2	19	2.465	0.651	43.919	2.115	0.347
m1	1	20	1.367	0.505	45.203	3.399	0.183
m9	3	18	11.977	0.062	51.072	9.268	0.010
m5	2	19	11.671	0.020	53.125	11.321	0.003
m8	4	17	20.664	0.008	57.421	15.617	0.000
m4	3	18	20.420	0.002	59.515	17.711	0.000
m6	3	18	20.420	0.002	59.515	17.711	0.000
m2	2	19	19.322	0.001	60.777	18.972	0.000
m10	4	17	29.932	0.000	66.689	24.884	0.000

k= number of independence claims; q= number of parameters

Table S9. Path coefficient estimates in the best-supported models for mammals and birds. Abbreviations: Body = body size (log); Brain = residual brain size; Newborn = residual newborn body size; BMR = residual basal metabolic rate; Ta = mean ambient temperature; Tb = mean body temperature.

Path	Estimate	95% confidence intervals
**Birds (best-supported models: m1 and m3, shown in Figure S2)**		
**Ta → Body**	**-0.150**	**(-0.222, -0.078)**
**Ta → BMR**	**-0.256**	**(-0.395, -0.118)**
Ta → Tb	0.097	(-0.047, 0.240)
Ta → Newborn	0.016	(-0.102, 0.134)
**Ta → Brain**	**-0.118**	**(-0.207, -0.028)**
**Body → BMR**	**0.258**	**(0.038, 0.479)**
Body → Tb	-0.177	(-0.401, 0.048)
**Body → Newborn**	**-0.307**	**(-0.514, -0.100)**
**Body → Brain**	**0.356**	**(0.180, 0.533)**
**BMR → Tb**	**0.156**	**(0.008, 0.305)**
BMR → Brain	0.050	(-0.030, 0.130)
Tb → Newborn	0.110	(-0.012, 0.232)
**Tb → Brain**	**0.113**	**(0.022, 0.203)**
**Newborn → Brain**	**0.161**	**(0.049, 0.273)**
**Mammals (best-supported model: m7, shown in Figure S2)**		
Ta → Body	-0.015	(-0.069, 0.040)
**Ta → BMR**	**-0.261**	**(-0.383, -0.138)**
Ta → Tb	-0.114	(-0.234, 0.006)
Ta → Newborn	0.015	(-0.020, 0.050)
Body → BMR	-0.017	(-0.184, 0.151)
Body → Tb	0.042	(-0.108, 0.193)
**Body → Newborn**	**-0.254**	**(-0.318, -0.190)**
**Body → Brain**	**-0.401**	**(-0.556, -0.246)**
**BMR → Tb**	**0.237**	**(0.118, 0.356)**
**Tb → Newborn**	**-0.041**	**(-0.080, -0.002)**
**Tb → Brain**	**0.127**	**(0.042, 0.212)**
**Newborn → Brain**	**0.959**	**(0.684, 1.234)**
Significant paths, defined as those with 95% confidence intervals not overlapping zero, are shown in bold.		

Table S10. Phylogenetically controlled mixed model in the R package *MCMCglmm* assessing the effect of body size, basal metabolic rate (BMR), newborn mass, body temperature (Tb) and ambient temperature (Ta) on brain size in birds and mammals.

	Posterior Mean	lower ▪ upper 95% CI	effective sample size	p MCMC
		**Birds (n = 177)**		
Intercept	-0.980	-1.979 ▪ -0.019	10000	0.052
**Body (log-10)**	**0.347**	**0.170 ▪ 0.526**	**10303**	**<0.001*****
**Newborn mass (residual)**	**0.169**	**0.062 ▪ 0.287**	**10000**	**0.001****
BMR (residual)	0.055	-0.023 ▪ 0.137	9083	0.174
**Body temperature (Tb)**	**0.113**	**0.025 ▪ 0.209**	**9621**	**0.021***
**Ambient temperature (Ta)**	**-0.115**	**-0.207 ▪ -0.026**	**9406**	**0.016***
		**Mammals (n = 240)**		
Intercept	1.543	-0.149 ▪ 3.216	9614	0.072
**Body (log-10)**	**-0.401**	**-0.569 ▪ -0.247**	**10000**	**<0.001*****
**Newborn mass (residual)**	**0.950**	**0.662 ▪ 1.225**	**10000**	**<0.001*****
BMR (residual)	0.024	-0.062 ▪ 0.110	10000	0.596
**Body temperature (Tb)**	**0.125**	**0.040 ▪ 0.217**	**9488**	**0.009****
Ambient temperature (Ta)	0.010	-0.067 ▪ 0.091	10000	0.810
		**Mammals (n = 240, with basal metabolic rate quotient[a])**		
Intercept	1.547	-0.124 ▪ 3.238	10000	0.070
**Body (log-10)**	**-0.400**	**-0.561 ▪ -0.241**	**10000**	**<0.001*****
**Newborn mass (residual)**	**0.954**	**0.684 ▪ 1.240**	**10391**	**<0.001*****
BMR quotient[a]	0.010	-0.069 ▪ 0.088	12298	0.804
**Body temperature (Tb)**	**0.127**	**0.037 ▪ 0.215**	**9300**	**0.006****
Ambient temperature (Ta)	0.004	-0.072 ▪ 0.084	10000	0.910

[a]Following Yegian et al.(6), BMR was scaled to an interspecific average that controls for variation in body size and environmental temperature.
Significant predictors are highlighted in bold.

Below (Tables S11–S15), we present analyses using relative brain mass and relative newborn mass as alternatives to log10-transformed data to address two potential concerns: (1) potential multicollinearity between newborn size and adult body size, and (2) the possibility that newborn sizes in ectotherms originate from specimens with highly deviant adult body mass. **Notably, all these analyses (Tables S11–S15) produced results identical to the original ones (Tables 1 and 2 in the main text, Supplementary Tables S3, S4, and S7).**

Within Classes

To address these concerns, we used residual brain mass and residual newborn mass, both derived from the body mass of each specimen. Residual offspring mass and residual brain mass were calculated using linear models in R base package that related log10-transformed body mass to log10-transformed newborn mass and brain mass separately for each class.

Among Classes

Due to the imbalance in sample sizes per class, we estimated residual brain mass and residual newborn mass using a phylogenetic generalized least squares model in the R package *phylolm* (7) across all vertebrates, deriving these values from each specimen's body mass.

Table S11. Phylogenetically controlled mixed model in the R package *MCMCglmm* assessing the effect of relative newborn mass on relative brain mass in each of the vertebrate classes with effective sample size.

	Posterior Mean	lower ▪ upper 95% CI	ESS	p MCMC
		**Lampreys (n = 14)**		
Intercept	0.068	-4.065 ▪ 4.267	10239	0.985
Body (log-10)	0.060	-0.334 ▪ 0.464	10000	0.657
Newborn mass (residual)	0.344	-0.384 ▪ 0.939	8274	0.371
		**Cartilaginous fishes (n = 120)**		
Intercept	-0.288	-1.468 ▪ 0.827	10000	0.618
**Body (log-10)**	**-0.195**	**-0.337 ▪ -0.046**	**5581**	**0.011***
**Newborn mass (residual)**	**0.228**	**0.061 ▪ 0.376**	**10000**	**0.005****
		**Ray-finned fishes (n = 210)**		
Intercept	-0.212	-1.691 ▪ 1.220	10000	0.779
**Body (log-10)**	**-0.342**	**-0.456 ▪ -0.228**	**9775**	**<0.001*****
Newborn mass (residual)	0.037	-0.096 ▪ 0.165	10289	0.591
		**Amphibians (n = 130)**		
Intercept	-0.281	-1.302 ▪ 0.807	10000	0.568
**Body (log-10)**	**-0.234**	**-0.426 ▪ -0.045**	**8825**	**0.022***
Newborn mass (residual)	0.102	-0.097 ▪ 0.306	9507	0.311
		**Reptiles (n = 190)**		
Intercept	0.120	-0.989 ▪ 1.266	10477	0.848
Body (log-10)	0.148	-0.044 ▪ 0.357	8821	0.147
Newborn mass (residual)	0.054	-0.066 ▪ 0.177	10000	0.375
		**Birds (n = 1136)**		
Intercept	-1.189	-1.914 ▪ -0.475	9628	0.001
**Body (log-10)**	**0.319**	**0.241 ▪ 0.389**	**10479**	**<0.001*****
**Newborn mass (residual)**	**0.250**	**0.200 ▪ 0.300**	**10000**	**<0.001*****
		**Mammals (n = 855)**		
Intercept	0.589	-0.820 ▪ 2.021	10000	0.415
**Body (log-10)**	**-0.783**	**-0.886 ▪ -0.678**	**10000**	**<0.001*****
**Newborn mass (residual)**	**0.515**	**0.408 ▪ 0.618**	**9290**	**<0.001*****

Significant predictors are highlighted in bold.

Table S12. Phylogenetically controlled mixed model in the R package *MCMCglmm* assessing the effect of temperature and relative newborn mass on relative brain mass across all vertebrates (n = 1059).

	Posterior Mean	lower ▪ upper 95% CI	ESS	p MCMC
Intercept	-1.039	-1.811 ▪ -0.239	10000	0.0090
**Body (log-10)**	**0.051**	**0.018 ▪ 0.083**	**10000**	**0.003****
**Newborn mass (residual, NM)**	**0.358**	**0.287 ▪ 0.437**	**9678**	**<0.001*****
**Body temperature (Tb)**	**0.231**	**0.164 ▪ 0.302**	**10000**	**<0.001*****
**NM × Tb**	**0.064**	**0.022 ▪ 0.107**	**10000**	**0.003****

Significant predictors are highlighted in bold.

Table S13. Phylogenetically controlled mixed model in the R package *MCMCglmm* assessing the effect of egg care on relative newborn mass (residual) in the classes.

	Posterior Mean	lower ▪ upper 95% CI	effective sample size	p MCMC
		**Cartilaginous fishes**		
**Egg abandoning vs live bearing and pre-hatching provisioning**				
Intercept	0.102	-0.564 ▪ 0.829	10000	0.782
**Body (log-10)**	**-0.191**	**-0.278 ▪ -0.101**	**10549**	**<0.001****
Egg care (*egg abandoning vs live bearing*)	0.352	-0.100 ▪ 0.801	10000	0.132
**Egg care (*egg abandoning vs pre-hatching provisioning*)**	**0.755**	**0.296 ▪ 1.212**	**10621**	**0.001****
**Live bearing vs pre-hatching provisioning**				
Intercept	0.672	-0.250 ▪ 1.667	9590	0.170
**Body (log-10)**	**-0.200**	**-0.298 ▪ -0.094**	**9415**	**<0.001*****
Egg care (*live bearing vs pre-hatching provisioning*)	0.385	-0.043 ▪ 0.809	10321	0.078
		**Ray-finned fishes**		
**Egg abandoning vs egg guarding and live bearing**				
Intercept	0.214	-1.167 ▪ 1.629	10000	0.763
**Body (log-10)**	**0.132**	**0.030 ▪ 0.232**	**10000**	**0.012***
Egg care (*egg abandoning vs egg guarding*)	0.202	-0.150 ▪ 0.566	10000	0.263
**Egg care (*egg abandoning vs live bearing*)**	**0.802**	**0.324 ▪ 1.249**	**10000**	**0.002****
**Egg guarding and live bearing**				
Intercept	-0.039	-2.542 ▪ 2.249	10000	0.976
**Body (log-10)**	**0.183**	**0.020 ▪ 0.352**	**10000**	**0.033***
**Egg care (*egg guarding vs live bearing*)**	**0.686**	**0.100 ▪ 1.263**	**10000**	**0.024***
		**Amphibians**		
**Egg abandoning vs egg guarding and live bearing**				
Intercept	-0.269	-0.930 ▪ 0.388	9942	0.409
**Body (log-10)**	**0.292**	**0.127 ▪ 0.454**	**9601**	**<0.001*****
**Egg care (*egg abandoning vs egg guarding*)**	**0.412**	**0.093 ▪ 0.725**	**10000**	**0.009****
Egg care (*egg abandoning vs live bearing*)	0.576	-0.017 ▪ 1.187	10000	0.064
**Egg guarding and live bearing**				
Intercept	0.293	-0.420 ▪ 0.983	10000	0.358

Body (log-10)	0.263	-0.024 ▪ 0.537	10000	0.074
Egg care (*egg guarding vs live bearing*)	-0.092	-0.804 ▪ 0.624	10000	0.786
		**Reptiles**		
**Egg abandoning vs egg guarding and live bearing**				
Intercept	-0.102	-0.518 ▪ 0.304	10000	0.614
Body (log-10)	0.001	-0.069 ▪ 0.070	10000	0.975
Egg care (*egg abandoning vs egg guarding*)	0.102	-0.063 ▪ 0.251	9720	0.192
Egg care (*egg abandoning vs live bearing*)	0.016	-0.143 ▪ 0.174	10000	0.833
**Egg guarding and live bearing**				
Intercept	-0.279	-0.708 ▪ 0.139	10703	0.172
**Body (log-10)**	**0.101**	**0.000 ▪ 0.206**	**10317**	**0.045***
Egg care (*egg guarding vs live bearing*)	-0.012	-0.241 ▪ 0.211	10000	0.910

Significant predictors are highlighted in bold.

Table S14. Phylogenetically controlled mixed model in the R package *MCMCglmm* assessing the effect of relative newborn mass on relative adult brain mass for various egg care categories.

	Posterior Mean	lower ▪ upper 95% CI	effective sample size	p MCMC
		**Cartilaginous fishes**		
**Egg abandoning (n = 26)**				
Intercept	0.260	-1.465 ▪ 1.948	10000	0.708
Body (log-10)	-0.047	-0.462 ▪ 0.390	9337	0.822
Newborn mass (residual)	0.220	-0.271 ▪ 0.676	8318	0.355
**Live bearing (n = 36)**				
Intercept	0.004	-1.877 ▪ 2.041	10000	0.999
**Body mass (log-10)**	**-0.502**	**-0.828 ▪ -0.161**	**10000**	**0.004****
Newborn mass (residual)	0.086	-0.209 ▪ 0.389	9122	0.568
**Pre-hatching provisioning (n = 58)**				
Intercept	-0.760	-2.597 ▪ 1.048	10000	0.395
Body mass (log-10)	-0.171	-0.393 ▪ 0.076	7660	0.153
**Newborn mass (residual)**	**0.247**	**0.030 ▪ 0.469**	**10000**	**0.028***
		**Ray-finned fishes**		
**Egg abandoning (n = 107)**				
Intercept	-0.805	-2.453 ▪ 0.876	10000	0.333
**Body mass (log-10)**	**-0.465**	**-0.616 ▪ -0.318**	**10000**	**<0.001****
Newborn mass (residual)	0.017	-0.170 ▪ 0.208	10000	0.858
**Egg guarding (n = 42)**				
Intercept	0.845	-1.510 ▪ 3.200	10000	0.479
**Body mass (log-10)**	**-0.374**	**-0.653 ▪ -0.116**	**9568**	**0.006****
Newborn mass (residual)	-0.025	-0.315 ▪ 0.303	9512	0.866
**Live bearing (n = 47)**				
Intercept	0.002	-2.153 ▪ 2.306	10088	0.994
**Body mass (log-10)**	**-0.204**	**-0.381 ▪ -0.038**	**10756**	**0.021***
**Newborn mass (residual)**	**0.235**	**0.013 ▪ 0.441**	**10000**	**0.031***
		**Amphibians**		
**Egg abandoning (n = 90)**				
Intercept	-0.537	-1.462 ▪ 0.464	10000	0.219
Body mass (log-10)	-0.208	-0.424 ▪ 0.017	9692	0.064
Newborn mass (residual)	0.000	-0.220 ▪ 0.221	10000	0.990
**Egg guarding (n = 25)**				

Intercept	0.265	-0.977 ▪ 1.502	10000	0.644
Body mass (log-10)	-0.256	-0.718 ▪ 0.229	10000	0.278
**Newborn mass (residual)**	**0.491**	**0.039 ▪ 0.961**	**9304**	**0.039***
		**Reptiles**		
**Egg abandoning (n = 77)**				
Intercept	-0.134	-1.725 ▪ 1.469	10000	0.865
Body mass (log-10)	0.163	-0.185 ▪ 0.502	10000	0.340
Newborn mass (residual)	-0.053	-0.260 ▪ 0.173	10000	0.624
**Egg guarding (n = 24)**				
Intercept	0.314	-1.031 ▪ 1.790	10000	0.636
Body mass (log-10)	-0.453	-1.312 ▪ 0.303	7597	0.263
**Newborn mass (residual)**	**0.529**	**0.037 ▪ 1.034**	**8765**	**0.041***
**Live bearing (n = 34)**				
Intercept	-0.074	-2.196 ▪ 1.918	10550	0.948
Body mass (log-10)	-0.119	-0.503 ▪ 0.284	7136	0.544
Newborn mass (residual)	0.057	-0.239 ▪ 0.370	9028	0.709
		**Birds**		
**Incubating birds (n = 1132)**				
Intercept	-1.145	-1.870 ▪ -0.435	10000	0.002
**Body (log-10)**	**0.322**	**0.246 ▪ 0.395**	**10000**	**<0.001*** **
**Newborn mass (residual)**	**0.254**	**0.204 ▪ 0.304**	**10193**	**<0.001*** **
		**Mammals (n = 855)**		
**All mammals (n = 855, similar model to Table S11)**				
Intercept	0.589	-0.820 ▪ 2.021	10000	0.415
**Body (log-10)**	**-0.783**	**-0.886 ▪ -0.678**	**10000**	**<0.001*** **
**Newborn mass (residual)**	**0.515**	**0.408 ▪ 0.618**	**9290**	**<0.001*** **

Significant predictors are highlighted in bold.

Table S15. Phylogenetically controlled mixed model in the R package *MCMCglmm* assessing the effect of temperature and relative newborn mass on relative brain mass within classes.

	Posterior Mean	lower ▪ upper 95% CI	effective sample size	p MCMC
		**Cartilaginous fishes (n = 118)**		
Intercept	-0.194	-1.391 ▪ 0.884	10000	0.729
**Body (log-10)**	**-0.196**	**-0.333 ▪ -0.045**	**5520**	**0.008****
**Newborn mass (residual)**	**0.200**	**0.047 ▪ 0.362**	**10000**	**0.012***
**Body temperature**	**0.250**	**0.068 ▪ 0.447**	**10000**	**0.008****
		**Ray-finned fishes (n = 159)**		
Intercept	-0.079	-2.092 ▪ 1.878	10000	0.934
**Body (log-10)**	**-0.329**	**-0.462 ▪ -0.213**	**10000**	**<0.001*****
Newborn mass (residual)	0.033	-0.110 ▪ 0.181	9543	0.657
Body temperature	0.132	-0.019 ▪ 0.303	9597	0.109
		**Amphibians (n = 41)**		
Intercept	0.101	-0.884 ▪ 1.166	9555	0.813
Body (log-10)	-0.065	-0.453 ▪ 0.316	8007	0.748
Newborn mass (residual)	0.210	-0.209 ▪ 0.648	10000	0.339
Body temperature	0.097	-0.350 ▪ 0.553	7872	0.673
		**Reptiles (n = 112)**		
Intercept	0.226	-0.944 ▪ 1.378	10000	0.704
Body (log-10)	-0.011	-0.295 ▪ 0.277	9639	0.943
Newborn mass (residual)	-0.029	-0.200 ▪ 0.151	10000	0.751
Body temperature	0.201	-0.033 ▪ 0.423	9507	0.085
		**Birds (n = 348)**		
Intercept	-1.112	-1.964 ▪ -0.282	10000	0.0102
**Body (log-10)**	**0.397**	**0.263 ▪ 0.528**	**10000**	**<0.001*****
**Newborn mass (residual)**	**0.255**	**0.156 ▪ 0.353**	**10000**	**<0.001*****
**Body temperature**	**0.104**	**0.033 ▪ 0.175**	**10000**	**0.005****
		**Mammals (n =274)**		
Intercept	1.391	-0.362 ▪ 2.927	9691	0.096
**Body (log-10)**	**-0.432**	**-0.580 ▪ -0.283**	**10000**	**<0.001*****
**Newborn mass (residual)**	**0.890**	**0.647 ▪ 1.145**	**10000**	**<0.001****
**Body temperature**	**0.127**	**0.042 ▪ 0.217**	**10000**	**0.005****

Significant predictors are highlighted in bold.

Table S16. Phylogenetically controlled mixed model in the R package *MCMCglmm* assessing the effect of internal (n = 11) and external (n = 175) fertilization on adult brain mass in ray-finned fishes. The fertilization modes were obtained from FishBase (2).

	Posterior Mean	lower ▪ upper 95% CI	effective sample size	p MCMC
**a). Effect on newborn mass (residual)**				
Intercept	0.222	-1.203 ▪ 1.659	10000	0.749
**Body mass (log-10)**	**0.112**	**0.007 ▪ 0.222**	**10000**	**0.041***
Fertilization (external vs internal)	0.343	-0.190 ▪ 0.812	10000	0.178
**b). Effect on brain mass (residual)**				
Intercept	0.073	-0.301 ▪ 0.469	10000	0.708
**Body mass (log-10)**	**-0.077**	**-0.108 ▪ -0.047**	**10000**	**<0.001*****
**Fertilization (external vs internal)**	**-0.161**	**-0.295 ▪ -0.030**	**9550**	**0.017***
**c). Effect on body mass (log-10)**				
Intercept	2.473	0.644 ▪ 4.143	10000	0.006
Fertilization (external vs internal)	0.576	-0.046 ▪ 1.224	10000	0.074

Significant predictors are highlighted in bold.

SI References

1. G. Katona, *et al.*, Evolution of reproductive modes in sharks and rays. *J Evol Biol* **36**, 1630–1640 (2023).
2. R. Froese, D. Pauly, FishBase. World Wide Web electronic publication. [Preprint] (2024).
3. A. I. Furness, I. Capellini, The evolution of parental care diversity in amphibians. *Nat Commun* **10**, 4709 (2019).
4. B. Halliwell, T. Uller, B. R. Holland, G. M. While, Live bearing promotes the evolution of sociality in reptiles. *Nat Commun* **8**, 2030 (2017).
5. M. Griesser, S. M. Drobniak, S. M. Graber, C. van Schaik, Parental provisioning drives brain size in birds. *Proc Natl Acad Sci U S A* **120**, e2121467120 (2023).
6. A. K. Yegian, *et al.*, Metabolic scaling, energy allocation tradeoffs, and the evolution of humans' unique metabolism. *Proceedings of the National Academy of Sciences* **121** (2024).
7. L. si T. Ho, C. Ane, A linear-time algorithm for gaussian and non-gaussian trait evolution models. *Syst Biol* **63**, 397–408 (2014).
